# Mechanobiologically Engineered Mimicry of Extracellular Vesicles for Improved Systemic Biodistribution and Anti‐Inflammatory Treatment Efficacy in Rheumatoid Arthritis

**DOI:** 10.1002/adhm.202500795

**Published:** 2025-08-09

**Authors:** Dahwun Kim, Hwira Baek, Su Yeon Lim, Min Sang Lee, Siyan Lyu, Jihyun Lee, Tun Naw Sut, Marta Gonçalves, Jeong Yi Kang, Joshua A. Jackman, Jin Woong Kim, Ji Hoon Jeong

**Affiliations:** ^1^ School of Pharmacy Sungkyunkwan University Suwon 16149 Republic of Korea; ^2^ School of Chemical Engineering Sungkyunkwan University Suwon 16149 Republic of Korea

**Keywords:** artificial extracellular vesicle, lipid/polymer hybrid liposome, membrane elasticity, systemic drug delivery, rheumatoid arthritis

## Abstract

Liposomal membrane elasticity is a controlling parameter in designing liposome‐based drug delivery systems and significantly affects biodistribution and biofunctional effects. Although extensively investigated in tumor models, the impact of liposomal membrane elasticity on rheumatoid arthritis (RA) remains underexplored. RA presents unique challenges, such as tortuous blood vessels, increased permeability, and chronic inflammation, which necessitate a specialized drug delivery strategy. This study aims to address these challenges by developing an engineered mimicry of extracellular vesicles (EVs) that is based on a lipid/polymer hybrid system incorporating poly(ethylene oxide)‐b‐poly(ε‐caprolactone)‐b‐poly(ethylene oxide) (PEO‐*b*‐PCL‐*b*‐PEO) to improve mechanical robustness and therapeutic performance.Tri‐ARTEX is developed as a lipid/polymer hybrid liposome encapsulating stem cell extract (CE) and microRNA (AntagomiR155), and tuned its membrane elasticity by varying the PEO‐*b*‐PCL‐*b*‐PEO fraction. Tri‐ARTEX exhibited enhanced cellular uptake in Raw 264.7 macrophages as the PEO‐b‐PCL‐b‐PEO fraction increases. However, semi‐elastic Tri‐ARTEX_8:2_ showed distinct biodistribution profiles and therapeutic effects in a murine collagen‐induced arthritis (CIA) model. Compared to its soft and rigid counterparts, semi‐elastic Tri‐ARTEX_8:2_ improved blood circulation, targeted accumulation in inflamed joints, and anti‐inflammatory efficacy. Our findings suggest that these mechanobiologically engineered liposomal EV mimics with regulated membrane elasticity provide new capabilities for designing drug nanocarriers for targeted RA therapy and help to address the unique pathophysiological challenges of RA.

## Graphical abstract

Triblock copolymer‐reinforced liposomal mimics of extracellular vesicles are engineered with tunable membrane elasticity. A semi‐elastic formulation (Tri‐ARTEX_8:2_) co‐encapsulating stem cell cytosolic extract and AntagomiR155 targets inflamed joints via the ELVIS effect, reprograms macrophages from M1 to M2, and markedly alleviates collagen‐induced arthritis in mice. This mechanical sweet spot optimizes uptake, biodistribution, and cytokine modulation, offering a scalable EV alternative for RA nanotherapy.

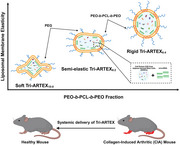

## 1 Introduction

Rheumatoid arthritis (RA) is a chronic systemic autoimmune disease that is characterized by joint swelling, redness, and severe inflammation of joints in the body.^[^ ^1^, ^2^ ^]^ These symptoms are caused by synovial extracellular matrix (ECM) dysregulation, infiltrating immune cell activation, pro‐inflammatory cytokine production, fibrotic mesh formation, and leaky vasculature development.^[^ ^2^, ^3^, ^4^ ^]^ Conventional RA treatments, including subcutaneous or intraarticular injections,^[^ ^5^ ^]^ ameliorate symptoms, but have limitations with short biological half‐lives, low bioavailability, and non‐specific targeting, often leading to severe systemic side effects and unwanted immune responses.^[^ ^6^, ^7^ ^]^ Effective systemic RA therapy necessitates a strategy for rapid drug distribution to target joints along with a safe and efficient delivery platform that can navigate the pathological microenvironment, increase systemic circulation time, and enhance tissue‐specific uptake.^[^ ^6^, ^8^, ^9^ ^]^ Designing such a platform is challenging for RA due to the abnormal synovial ECM and low cellular internalization capacity, which create biological and cellular barriers to drug delivery.^[^ ^3^, ^10^, ^11^ ^]^ The inflamed joints in RA exhibit unique pathophysiological characteristics such as tortuous blood vessels, increased permeability, and an inflammatory microenvironment. Extravasation through leaky vasculature and subsequent inflammatory cell‐mediated sequestration (ELVIS) can also be found in the inflamed joints in RA, which is similar to the enhanced permeability and retention (EPR) phenomenon in solid tumors.^[^ ^12^, ^13^, ^14^ ^]^ Thus, a drug carrier capable of overcoming these barriers and exploiting the extravasation through leaky vasculature and subsequent inflammatory cell‐mediated sequestration (ELVIS) effect is crucial for developing an effective and safe systemic RA therapy.

Extracellular vesicles (EVs) have emerged as potential cell‐free therapeutics for many diseases, including RA.^[^ ^15^, ^16^ ^]^ These cell‐derived lipid nanoparticles facilitate intercellular communication and modulate biological responses by delivering bioactive molecules.^[^ ^17^, ^18^, ^19^ ^]^ However, the clinical application of EV‐based therapy faces challenges in standardizing large‐scale production, ensuring high purity, and maintaining structural integrity during encapsulation and surface modification processes.^[^ ^20^ ^]^ Indeed, the encapsulation of therapeutic agents and membrane surface engineering may compromise the structural integrity of EVs, decreasing their stability and biological performance in blood.^[^ ^21^, ^22^ ^]^ These limitations call for alternative strategies that exploit the beneficial features of EVs while ensuring reproducibility and scalability.

Liposomes, which are artificial lipid bilayer vesicles ranging from 20–1000 nm in diameter, have long been employed as drug delivery platforms due to their excellent biocompatibility, low immunogenicity, and facile tunability of physicochemical properties.^[^ ^23^ ^]^ Numerous studies have modified liposomal properties such as particle size, surface charge, and surface absorption in order to enhance blood circulation and targeted cellular uptake.^[^ ^24^, ^25^, ^26^, ^27^ ^]^ Despite these advances, the role of liposomal membrane elasticity in modulating systemic circulation and target accumulation remains underexplored, particularly in the context of RA. Early studies on nanoparticle elasticity have shown that mechanical properties significantly affect nanoparticle behavior in biological systems. For example, hard nanoparticles exhibit enhanced cellular uptake due to lower required membrane tension, while soft nanoparticles benefit from prolonged circulation and increased target cell internalization due to reduced phagocytosis.^[^ ^28^, ^29^, ^30^, ^31^, ^32^ ^]^ For RA treatment, modulating liposomal membrane elasticity could help to address specific challenges related to the fibrotic synovial ECM and inflammatory microenvironment, potentially enhancing drug accumulation in inflamed joints and improving therapeutic efficacy.

**Figure 1:**
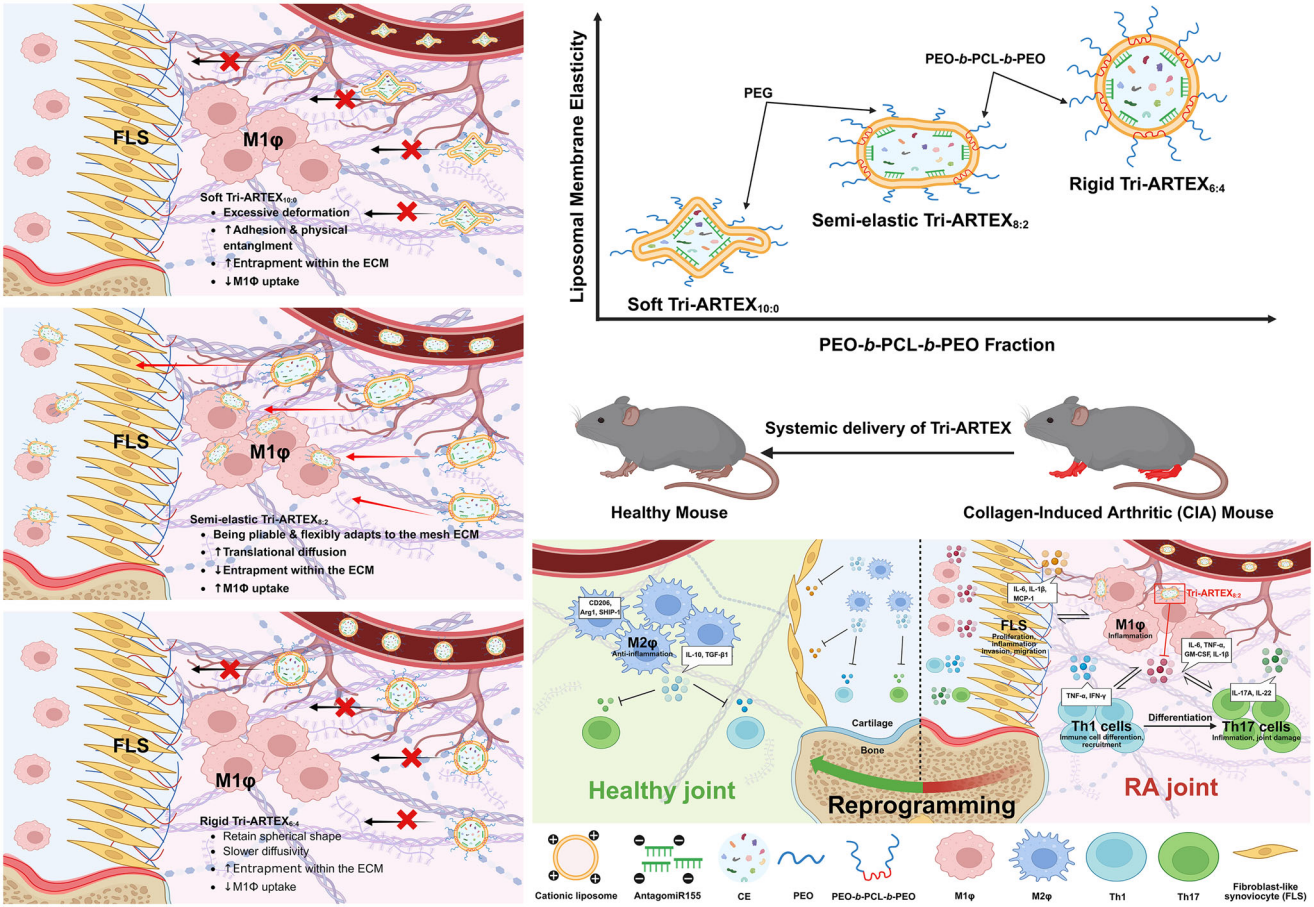
Schematic representation of the liposomal membrane elasticity‐mediated systemic delivery of Tri‐ARTEXs in RA. Liposomal membrane elasticity increases with increasing PEO‐*b*‐PCL‐*b*‐PEO. Soft Tri‐ARTEX_10:0_ exhibits excessive deformation, leading to increased adhesion and entrapment within the fibrotic ECM of the synovial joint. Rigid Tri‐ARTEX_6:4_ retains a spherical shape but exhibits slower diffusivity. Semi‐elastic Tri‐ARTEX_8:2_, being pliable, undergoes shape adaptation that promotes translational diffusion, minimizes ECM entrapment, and enhances targeted cellular internalization.

In this study, we hypothesized that fine‐tuning liposomal membrane elasticity using a lipid/polymer hybrid system with poly(ethylene oxide)‐*b*‐poly(ε‐caprolactone)‐*b*‐poly(ethylene oxide) (PEO‐*b‐*PCL‐*b*‐PEO) could enhance systemic biodistribution and anti‐inflammatory effects in RA (**Figure** **1**). By modifying the mechanical properties of EVs, such as elasticity, with PEO‐*b*‐PCL‐*b*‐PEO, we developed Tri‐ARTEX (triblock copolymer‐incorporated artificial extracellular vesicle mimicry), which is a lipid/polymer hybrid liposome encapsulating stem cell extract (CE) and antago‐microRNA‐155 (Antagomir155) to modulate membrane elasticity and improve mechanical robustness and biological versatility. This mechanobiologically engineered mimicry of EVs aims to address the limitations of EV therapy by providing a reproducible, scalable platform with controlled membrane properties, offering new capabilities to design drug nanocarriers for targeted RA therapy.

## 2 Results

### 2.1 Characterization of Tri‐LIPs and Tri‐ARTEXs

**Figure 2:**
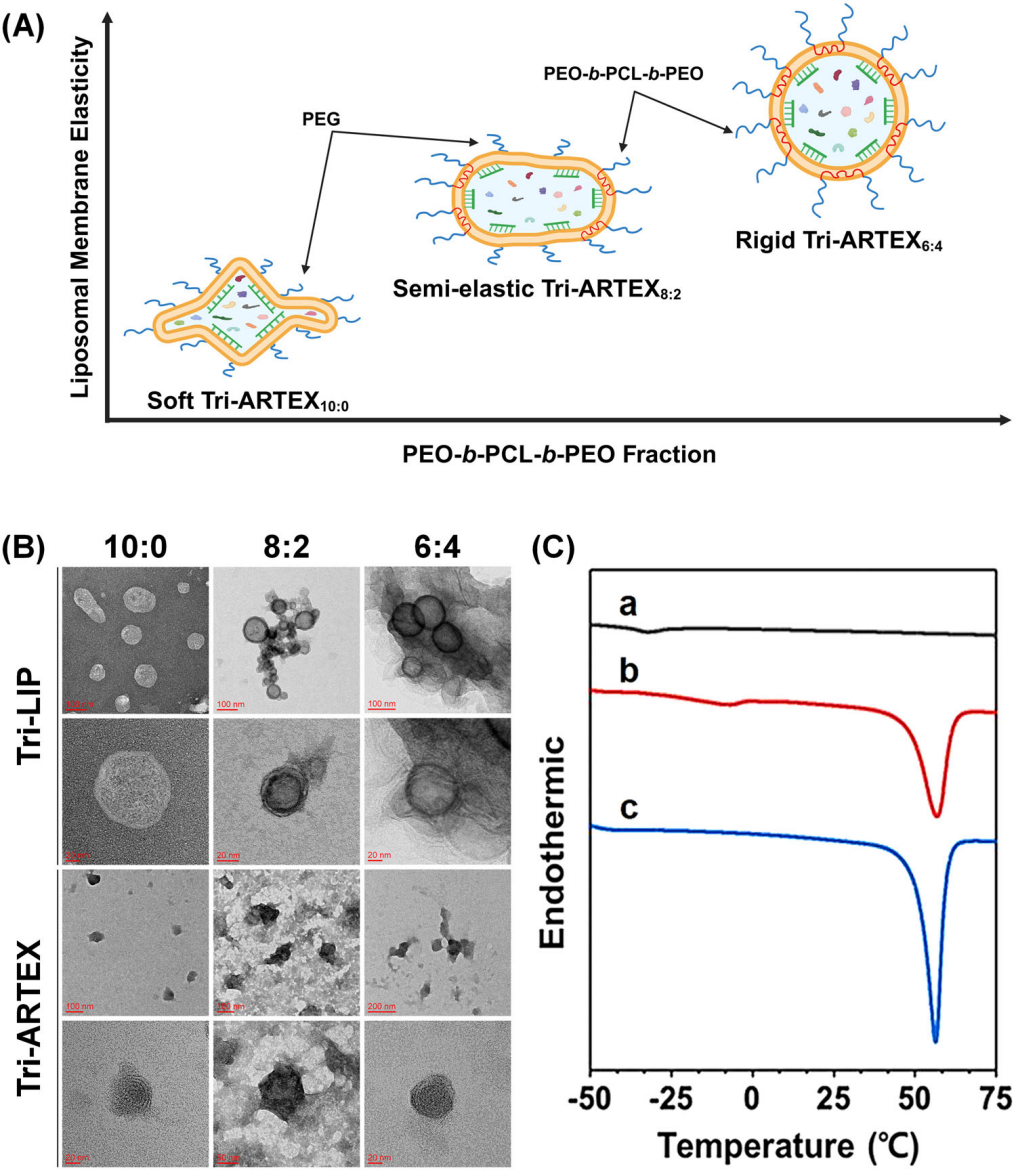
A) Schematic illustration for controlling the liposomal membrane elasticity of Tri‐LIPs with different PEO‐*b*‐PCL‐*b*‐PEO fractions. B) Prepared Tri‐LIPs and Tri‐ARTEXs were examined with transmission electron microscopy to assess their size and structural integrity. C) DSC thermograms of dried lipids, polymer, and lipid/polymer films are as follows: (a) DOPE/DOTAP (4/5, w/w), (b) a mixture of DOPE/DOTAP and PEO‐*b*‐PCL‐*b*‐PEO (8/2, w/w), and (c) PEO‐*b*‐PCL‐*b*‐PEO only.

We designed a liposome‐based EV mimicry with tunable membrane elasticity by co‐assembling PEO‐*b*‐PCL‐*b*‐PEO triblock copolymer with phospholipid molecules (Tri‐LIPs). Due to the high crystallinity of the PCL middle block, the membrane elasticity of the liposome could be enhanced, and the stability of Tri‐LIPs was also increased by the PEGylation effect, as illustrated in **Figure** **2**A.^[^ ^33^, ^34^ ^]^ Various Tri‐LIP formulations were prepared with ratios of phospholipids to PEO‐*b*‐PCL‐*b*‐PEO of 10:0, 8:2, and 6:4, and the surface density of PEG chains on the Tri‐LIPs was kept constant by adjusting the co‐assembly amount of PEG5K‐DOPE, as shown in Figure S1 and Table S1 (Supporting Information). Each Tri‐LIP formulation that encapsulates CE and microRNA 155 inhibitor (AntagomiR155) is described as Tri‐ARTEX. The characteristics of the Tri‐LIP and Tri‐ARTEX samples prepared in this study are summarized in **Table** **1**. Particle sizes and PDI values of the prepared Tri‐LIPs were found to be in a narrow range of ≈58–87 nm in diameter and 0.142–0.202, respectively. The positively charged Tri‐LIP characteristics were attributed to cationic DOTAP, which is useful for encapsulating negatively charged nucleic acid through electrostatic interactions.^[^ ^35^, ^36^, ^37^ ^]^ Encapsulation of CE and AntagomiR155 not only increased Tri‐ARTEX particle sizes but also changed their surface charges to slightly negative. In addition, the incorporation of 5 kDa PEG on the liposomal surface may also contribute to moderate colloidal stability sufficient to minimize complement activation and reticuloendothelial system (RES) clearance.^[^ ^38^, ^39^, ^40^ ^]^ Their PDI values remained less than 0.3, indicating that Tri‐ARTEXs are in an acceptable range of size and PDI values for targeting RA through the ELVIS effect.^[^ ^12^, ^41^ ^]^ The liposomal morphology of Tri‐LIP and Tri‐ARTEX was confirmed by TEM analysis (Figure 2B). The Tri‐LIPs and Tri‐ARTEXs exhibited a spherical shape and had no significant differences in morphology regardless of the PEO‐*b*‐PCL‐*b*‐PEO fraction. The coefficient of variation (COV%) was calculated to assess the repeatability of three different Tri‐ARTEX formulations. All formulations demonstrated COVs below 5% for particle size, PDI, zeta potential, loading content, and encapsulation efficiency, indicating high consistency and reproducibility across all Tri‐ARTEX formulations (Table S2, Supporting Information). A COV% below 5% is generally considered indicative of high reproducibility and acceptable variability in liposomal formulations.^[^ ^42^ ^]^ Achieving precise control of particle size through sonication^[^ ^43^, ^44^, ^45^ ^]^ in the Tri‐ARTEX formulation process was crucial for obtaining a size range similar to previously tested liposome formulations that liposomes within the 100–200 nm size range, slightly negatively charged, and incorporating 5 kDa PEG exhibit enhanced circulation time and targeted accumulation in inflamed joints of RA mouse models.^[^ ^46^ ^]^ The formulation method highlights the importance of optimizing particle size in the present design and motivates further exploration of the effects of liposomal membrane elasticity.

**Table 1:** Characterization of Tri‐LIPs and Tri‐ARTEXs with different PEO‐*b*‐PCL‐*b*‐PEO fractions.

	Particle diameter (nm) ^b)^		PDI ^b)^		Zeta potential (mV) ^b)^		LC (%) ^b)^		EE (%) ^b)^	
	Tri‐LIP	Tri‐ARTEX	Tri‐LIP	Tri‐ARTEX	Tri‐LIP	Tri‐ARTEX	CE	AntagomiR155	CE	AntagomiR155
10:0 ^a)^	59.98±1.91	113.3±4.91	0.195±0.007	0.188±0.029	3.03±0.40	−4.20±0.12	37.18±0.59	7.53±0.02	55.76±0.89	57.76±0.18
8:2 ^a)^	48.29±3.90	111.5±4.02	0.185±0.012	0.219±0.023	5.40±0.33	−4.34±0.08	38.50±0.42	7.38±0.03	57.75±0.62	56.55±0.23
6:4 ^a)^	86.25±0.63	135.6±31.6	0.159±0.017	0.239±0.033	13.5±1.91	−7.40±0.43.	40.62±0.73	8.43±0.01	60.93±1.10	64.61±0.09

For three Tri‐ARTEX preparations, LC, drug loading content;

EE, encapsulation efficiency;

^a)^ Composition ratio of phospholipids (DOTAP and DOPE) vs. PEO‐*b*‐PCL‐*b*‐PEO;

^b)^ Data are represented as mean ± SD from three independent experiments (n = 3).

The encapsulation efficiency (EE) of Tri‐ARTEXs in different formulations was quantified by measuring the amount of encapsulated CE and AntagomiR155 at ≈55–61% and 57–65%, respectively. The measured EE values were largely independent of the PEO‐*b*‐PCL‐*b*‐PEO fraction, demonstrating good control over the loaded drug amount across the different formulations and enabling us to focus on how changes in liposomal membrane elasticity affect biological functionalities. To investigate the lipid phase properties of Tri‐LIPs in the presence of PEO‐*b*‐PCL‐*b*‐PEO, the thermal behavior of each component of Tri‐LIPs, as well as the dried film of Tri‐LIPs, was characterized by DSC analysis (Figure 2C, Figure S2, Supporting Information). PEO‐*b*‐PCL‐*b*‐PEO exhibited a high melting peak at 56.4 °C and heating energy of 85.5 J g^−1^ due to its intrinsic crystallinity.^[^ ^47^, ^48^ ^]^ Even after mixing DOPE/DOTAP with PEO‐*b*‐PCL‐*b*‐PEO, only a slight lifting of the melting peak of PEO‐*b*‐PCL‐*b*‐PEO was observed while retaining heating enthalpy. This notable thermal property of the Tri‐LIP lipid phase seems to be attributed to the enhanced modulus of the liposomal membrane, which is achieved by the lateral position of the PCL block of PEO‐*b*‐PCL‐*b*‐PEO within the lipid bilayer.^[^ ^33^ ^]^ We further investigated the dispersion stability of Tri‐LIPs under near‐physiological conditions. All Tri‐LIPs were immobilized with Texas‐Red DHPE in the hydrophobic lipid bilayer and incubated with fetal bovine serum (FBS, 10% v/v) in PBS for 48 h. We observed that the fluorescence intensity of all Tri‐LIP formulations remained constant, implying that the vesicular membrane remained intact without significant degradation or aggregation (Figure S3, Supporting Information). We further evaluated the serum stability of AntagomiR155 encapsulated within Tri‐LIPs under physiological conditions. Agarose gel electrophoresis analysis revealed that following 72 h incubation with 50% (v/v) FBS at 37 °C, all Tri‐LIP formulations preserved the encapsulated AntagomiR155 (Figure S4, Supporting Information). These findings confirm that Tri‐LIPs protect against nuclease degradation during blood circulation, enabling targeted delivery of intact AntagomiR155 to inflammatory sites.

Western blotting analysis characterized the EV‐mimetic properties of the Tri‐ARTEX formulation using common EV marker proteins, including apoptosis‐linked gene‐2‐interacting protein X (ALIX), heat shock protein 70 kDa (Hsp70), and CD63 (Figure S5, Supporting Information). Both Tri‐ARTEX and CE showed significant ALIX and Hsp70 levels, but rather modest CD63 inclusion. On the other hand, the native EVs showed significant amounts of CD63 but low levels of ALIX and Hsp70. This distinct marker profile indicates that, although the membrane composition of Tri‐ARTEX differs from that of natural EVs, it biomimetically encapsulates CE components and mimics EV‐like biological properties. These results support the potential of Tri‐ARTEX as a functional EV‐mimetic system. Motivated by the self‐assembly of Tri‐ARTEXs and Tri‐LIPs into spherical nanoparticles and the EV‐mimetic properties of Tri‐ARTEX, the biophysical and biological analysis of Tri‐LIPs assessed how the PEO‐*b*‐PCL‐*b*‐PEO fraction influences nanomechanical properties and contributes to improved carrier design.

### 2.2 Tunability of Tri‐LIP liposomal membrane with PEO‐*b*‐PCL‐*b*‐PEO

To evaluate the mechanical strength of Tri‐LIP lipid bilayer membranes, we conducted QCM‐D measurements to characterize Tri‐LIP nanoparticle adsorption onto a titania‐coated sensor surface. In QCM‐D experiments, the resonance frequency (Δf) and energy dissipation (ΔD) shifts associated with Tri‐LIP adsorption onto an oscillating, titania‐coated sensor chip were tracked temporally and reflect the mass and viscoelastic properties, respectively, of the adsorbed lipid/polymer molecules within the Tri‐LIPs along with hydrodynamically coupled solvent inside and between the adhered Tri‐LIPs.^[^ ^49^ ^]^ Depending on the interplay of the nanoparticle‐surface interaction strength and the Tri‐LIP membrane bending energy, adhered Tri‐LIPs undergo different degrees of shape deformation, and thus variations in adlayer properties can provide insight into the relative mechanical strength of Tri‐LIP membranes.^[^ ^50^, ^51^ ^]^

**Figure 3:**
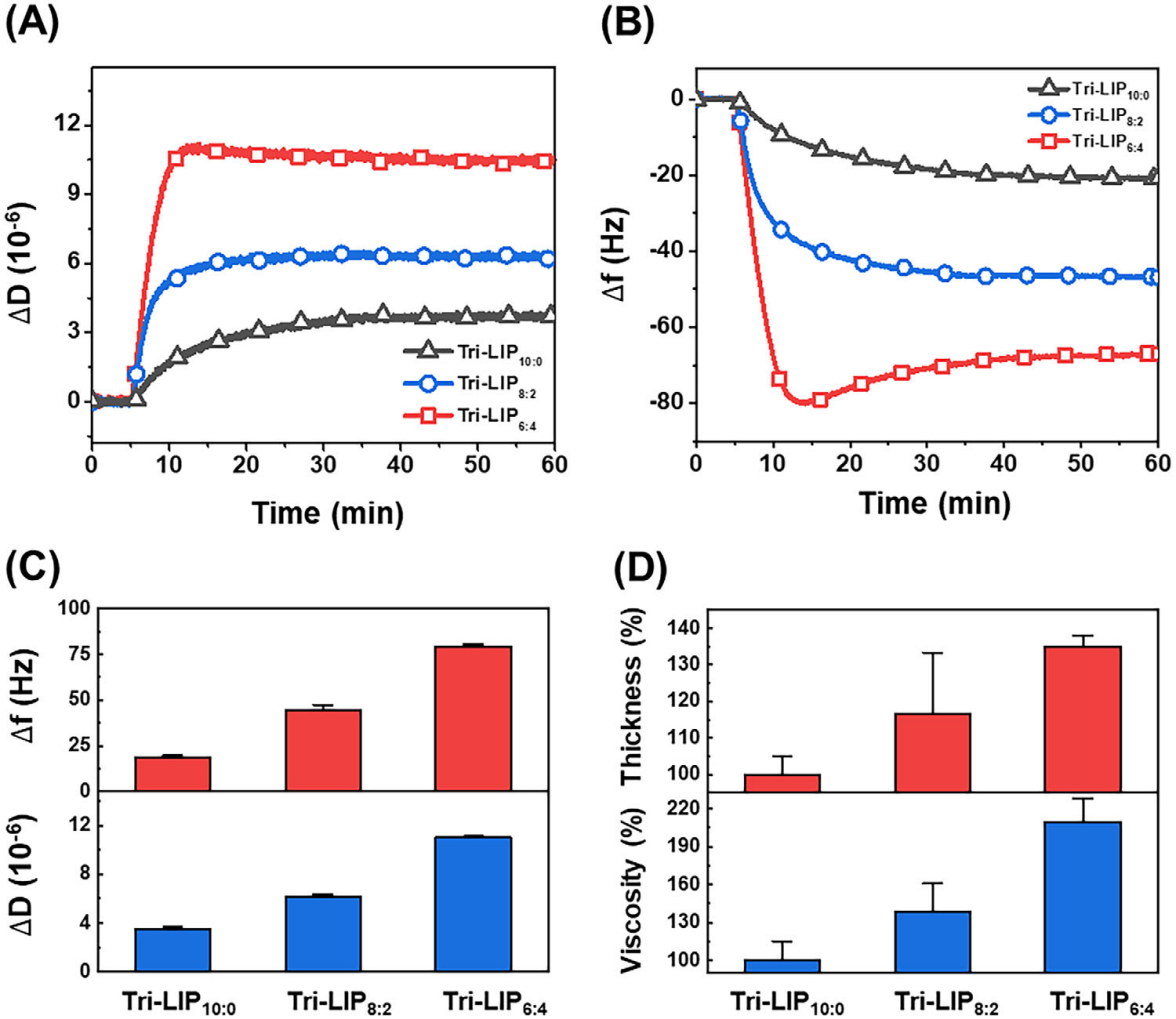
Time‐resolved quartz crystal microbalance‐dissipation (QCM‐D) measurement responses for A) resonance frequency (Δf) and B) energy dissipation (ΔD) signals upon Tri‐LIP addition to titania‐coated sensor chip surfaces. Samples were injected at *t*=5 min after initial stabilization in equivalent buffer. C) Magnitudes of final Δf and ΔD shifts at saturation. D) Thickness and viscosity of Tri‐LIP adlayers. Data are reported as relative percentage values, and larger values indicate greater mechanical strength of the Tri‐LIP membranes.

For QCM‐D measurements, all Tri‐LIP samples were prepared in 10 mm Tris buffer (pH 7.5) with 150 mm NaCl and were added to the titania‐coated sensor chip at *t* = 5 min after establishing baseline signals in equivalent buffer solution. A significant decrease in the Δf signal and an increase in the ΔD signal indicated that all Tri‐LIP samples adsorbed favorably onto the titania surface, and the measurement signals eventually stabilized, indicating adsorption saturation (**Figure** **3**A,B). Notably, larger maximum Δf and ΔD signals at saturation occurred for Tri‐LIP samples with greater PEO‐*b*‐PCL‐*b*‐PEO fraction, which suggests less shape deformation and greater mechanical strength (Figure 3C). The corresponding adsorption kinetics provided additional supporting evidence of variations in mechanical strength depending on the Tri‐LIP composition. In particular, monotonic adsorption kinetics were observed for Tri‐LIP_10:0_ and Tri‐LIP_8:2_, whereas the time‐resolved Δf signal for Tri‐LIP_6:4_ showed overshoot behavior that is characteristic of rigid nanoparticle attachment^[^ ^52^ ^]^ and implies that Tri‐LIP_6:4_ is more rigid than the other samples.

By applying the extended Voigt viscoelastic model,^[^ ^53^ ^]^ the effective film thickness of the Tri‐LIP adlayer could be fitted from the overtone‐dependent QCM‐D data and tended to increase by ≈35% at greater PEO‐*b*‐PCL‐*b*‐PEO fractions, signifying that polymer incorporation increases membrane bending energy to resist adsorption‐related deformation (Figure 3D, Figure S6, Supporting Information). In addition, the effective viscosity of the Tri‐LIP adlayer tended to increase by around twofold at greater PEO‐*b*‐PCL‐*b*‐PEO fractions, which is also consistent with the membrane strengthening effect.^[^ ^54^ ^]^ The adlayer thickness and viscosity were largest for adhered Tri‐LIP_6:4_ nanoparticles, supporting that its lipid bilayer membrane has the highest mechanical strength among the tested Tri‐LIP compositions.

### 2.3 Enhanced Cellular Uptake of Membrane Elasticity‐Controlled Tri‐LIPs

**Figure 4:**
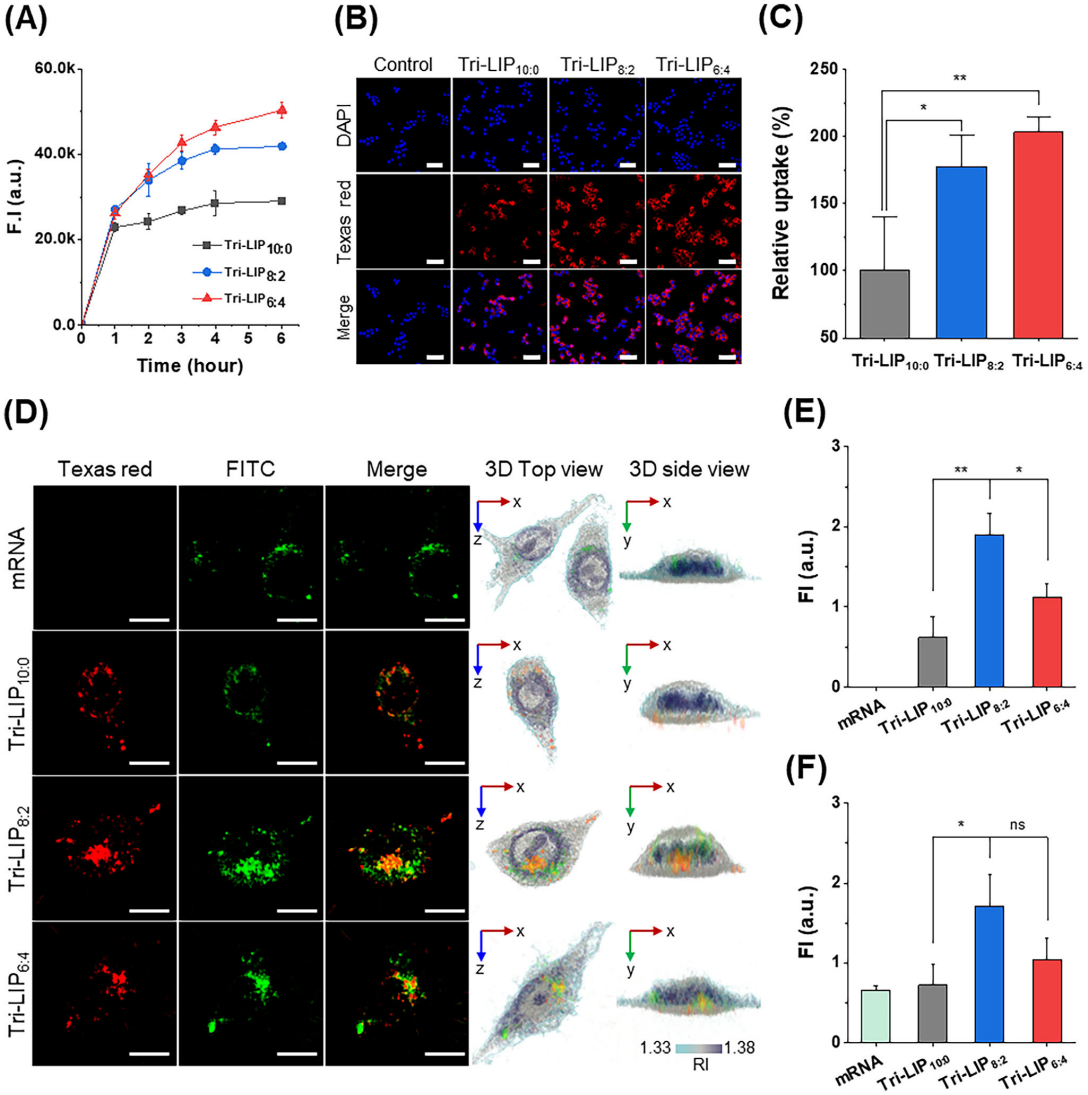
A) Cellular uptake of Tri‐LIPs as a function of incubation time. B) CLSM visualization and C) quantification of relative cellular uptake of Tri‐LIPs in Raw 264.7 cells after 4 h incubation. Texas Red‐DHPE was conjugated with each Tri‐LIP (red), and cell nuclei were stained with DAPI (blue). Scale bars are 50 µm. (n = 4 in each group, ^*^ *p* < 0.05, ^**^ *p* < 0.005 compared to Tri‐LIP10:0, one‐way ANOVA). D) 3D holotomography and fluorescence images of single macrophages, revealing the intracellular fate of FITC‐labeled free mRNA or mRNA‐loaded Tri‐LIPs. Scale bars are 10 µm. Quantitative analysis of E) Texas red fluorescence intensity demonstrating differential liposomal uptake and F) FITC fluorescence intensity indicating mRNA delivery efficiency across formulations. (n = 3 in each group, ^*^ *p* < 0.05, ^**^ *p* < 0.005 compared to Tri‐LIP8:2, one‐way ANOVA).

Liposomes with specific physicochemical properties, such as well‐controlled particle size, chemical composition, surface functionality, and/or mechanical stability, can not only pass through various biological barriers, including the blood‐brain barrier (BBB), but also protect their cargos and deliver them accurately to the target site.^[^ ^41^, ^55^ ^]^ LPS‐activated Raw 264.7 macrophages polarized to the M1 phenotype served as our inflammatory disease model for investigating Tri‐LIP mechanical property effects on cellular interactions (Figure S7, Supporting Information).^[^ ^41^, ^55^ ^]^ Tri‐LIPs demonstrated no cytotoxicity up to 50 µg mL^−1^ in Raw 264.7 cells (Figure S8, Supporting Information). Texas Red‐DHPE‐labeled Tri‐LIP uptake increased directly with PEO‐b‐PCL‐b‐PEO fraction, with Tri‐LIP_6:4_ delivering maximum cellular signal. PEO‐b‐PCL‐b‐PEO fraction increases enhanced cellular association dramatically, producing up to 174% higher uptake (**Figure** **4**A). Quantitative confocal laser scanning microscopy (CLSM) confirmed these trends across cell populations, revealing the highest fluorescence intensity for Tri‐LIP_6:4_, followed by Tri‐LIP_8:2_, then Tri‐LIP_10:0_, conclusively demonstrating that increased nanoparticle rigidity drives more efficient cellular association (Figure 4B,C).

High‐resolution 3D holotomography combined with fluorescence imaging distinguished membrane‐associated from internalized liposomes and revealed Tri‐LIP intracellular fate at single‐cell resolution. Texas‐red labeling of Tri‐LIPs and FITC‐conjugated mRNA enabled simultaneous component tracking throughout cellular processing. All formulations demonstrated clear Tri‐LIP‐mRNA co‐localization, confirming robust cargo retention within the liposomal carriers (Figure 4D). Quantitative orthogonal projection analysis revealed unexpected efficacy patterns, with Tri‐LIP_8:2_ achieving significantly higher intracellular accumulation of both lipid carrier (Figure 4E) and mRNA cargo (Figure 4F) compared to either the more compliant Tri‐LIP_10:0_ or more rigid Tri‐LIP_6:4_ formulations, establishing an optimal mechanical property threshold for intracellular delivery.

Combined cellular uptake analyses revealed critical mechanical property thresholds for effective internalization. Despite strong population‐level cellular association, 3D imaging demonstrated that rigid Tri‐LIP_6:4_ particles predominantly remained trapped at cell membranes or within intercellular spaces rather than achieving cytoplasmic delivery. Conversely, the highly compliant Tri‐LIP_10:0_ formulation undergoes significant shape deformation during endocytosis, necessitating substantially greater adhesion energy to initiate membrane wrapping,^[^ ^41^, ^55^ ^]^ thereby compromising internalization efficiency. Tri‐LIP_8:2_ formulations, exhibiting optimal mechanical properties, maintain sufficient rigidity for effective membrane interaction while preserving adequate flexibility to complete endocytosis and achieve superior intracellular delivery,^[^ ^41^, ^55^ ^]^ establishing a mechanical sweet spot for nanocarrier design that maximizes therapeutic payload delivery.

### 2.4 Effects of Membrane Elasticity on Tri‐LIP Biodistribution in CIA Mice

**Figure 5:**
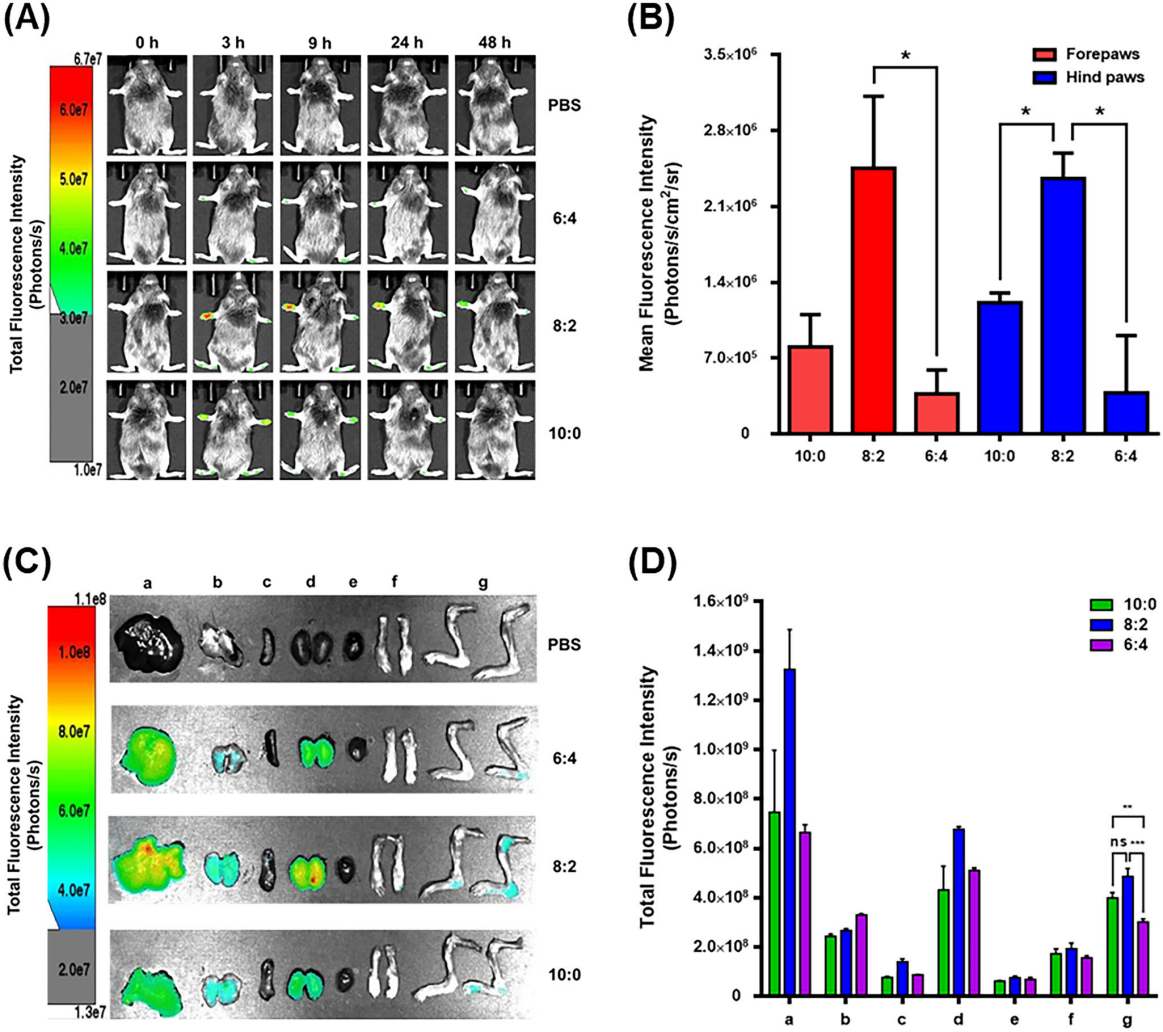
Effects of liposomal membrane elasticity of Tri‐LIPs on in vivo biodistribution in CIA mice. IV injection of PBS and Cy5.5‐labled Tri‐LIP preparations (10:0, 8:2, 6:4) into DBA1/J mice of RA model. A) CIA mice were imaged at intervals as follows: 0, 3, 9, 24, 48 h. A scale of the radiance efficiency is presented to the right. B) Quantitative analysis of Tri‐LIP preparations at 3 h. The mean fluorescence intensity was quantified by using Aura. C) Organs were excised after 3 h. A scale of the radiance efficiency is presented to the right; a. liver, b. lung, c. spleen, d. kidney, e. heart, f. front paws, g. hind paws. D) The total fluorescence intensity of liposome retention by each organ was quantified using Aura. Data are mean ± SEM (n = 5, ns: *p* > 0.05, ^*^ *p* ≤ 0.05, ^**^: *p* ≤ 0.01, ^***^: *p* ≤ 0.001 vs Tri‐ARTEX_8:2_).

Biodistribution is a critical design parameter for nanocarriers upon systemic circulation because it impacts the in vivo therapeutic efficacy of nanoparticles in RA.^[^ ^15^ ^]^ The role of liposomal membrane elasticity in biodistribution was investigated using Cy5.5‐labeled Tri‐LIPs injected into collagen‐induced arthritis (CIA) mice. In vivo imaging revealed that Tri‐LIP_8:2_ achieved the highest accumulation in inflamed paws, persisting for 48 h, while Tri‐LIP_10:0_ and Tri‐LIP_6:4_ exhibited shorter circulation times and lower accumulation (**Figure** **5**A). At 3 h post‐injection, the mean fluorescence intensity of Tri‐LIP_8:2_ was significantly higher than that of Tri‐LIP_10:0_ and Tri‐LIP_6:4_ (Figure 5B). Ex vivo imaging of major organs and inflamed joints at 3 h post‐injection further confirmed that Tri‐LIP_8:2_ efficiently targeted inflamed joints and paws, whereby a large number of activated synoviocytes and proinflammatory macrophages were infiltrated (Figure 5C,D). Tri‐LIP_8:2_ with intermediate elasticity efficiently targeted inflamed joints, overcoming the tortuous blood vessels and the fibrotic synovial ECM, while more soft and rigid counterparts were less effective in the RA‐related microenvironment. Although biodistribution and accumulation were dynamically monitored via in vivo and *ex vivo* imaging, future studies involving blood sampling and quantitative analysis of nanoparticle concentrations from the body system will incorporate comprehensive pharmacokinetic evaluations, including circulation half‐life and site‐specific drug release kinetics, to further elucidate the influence of liposomal membrane elasticity on in vivo delivery dynamics.

### 2.5 Effects of elasticity on in vivo therapeutic efficacy of Tri‐ARTEX in CIA mice

To evaluate the therapeutic effects of elasticity‐tuned Tri‐ARTEX, CIA mice were treated with PBS, MTX, or different Tri‐ARTEX formulations via intravenous injection, following the regimen illustrated in **Figure** **6**A. Over a 3‐week period starting on day 21 post‐immunization, body weights, clinical arthritic scores, and paw thickness were monitored. Significant body weight recovery was observed in the Tri‐ARTEX_8:2_ group, showing similar results to the Sham group, indicating a reduction in disease severity. By contrast, Tri‐ARTEX_10:0_ and Tri‐ARTEX_6:4_ showed moderate improvements comparable to the MTX group (Figure 6B‐D). Arthritic scores and paw thickness were markedly lower in all Tri‐ARTEX‐treated groups compared to the CIA and CE+AntagomiR155 groups, indicating the therapeutic potential of the formulations. Notably, Tri‐ARTEX_8:2_ exhibited the most significant reduction in swelling and redness, with clinical scores and paw thickness nearing those of the Sham group (Figure 6C,D). Morphological and histological examinations revealed extensive inflammation, synovial hyperplasia, bone erosion, and cartilage destruction in the CIA and CE+AntagomiR155 groups (Figure 6E,F). However, all Tri‐ARTEX‐treated groups showed significantly reduced histopathological signs of RA, with Tri‐ARTEX_8:2_‐treated mice displaying minimal synovial infiltration, intact bone structure, and cartilage preservation (Figure 6F). Tri‐ARTEX_10:0_ and Tri‐ARTEX_6:4_ groups demonstrated mild to moderate inflammation and structural damage, demonstrating partially enhanced bone and cartilage erosions with mild or moderate pannus invasion. Quantitative histological scoring confirmed that Tri‐ARTEX_8:2_ significantly lowered inflammation, synovial hyperplasia, pannus formation, and bone/cartilage erosion scores compared to other Tri‐ARTEX groups (Figure 6G).

Figure 6Effects of liposomal membrane elasticity of Tri‐ARTEXs on in vivo therapeutic efficacy in CIA mice. A) Therapeutic regimen used for the MTX and Tri‐ARTEXs with different PEO‐*b*‐PCL‐*b*‐PEO fractions. B) Body weight monitoring. The severity of arthritic incidence was determined by C) an arthritic scoring system and D) the paw thickness of the CIA mouse. Data is represented in mean ± SEM (n = 7, ns: *p* > 0.05, ^*^: *p* ≤ 0.05, ^***^: *p* ≤ 0.001, ^****^: *p* ≤ 0.0001 versus CIA; NS: *p* > 0.05, #: *p* ≤ 0.05, ##: *p* ≤ 0.01 versus Tri‐ARTEX_8:2_. E) Representative images of the sham and the CIA mice treated with PBS, MTX, CE+AntagomiR155, Tri‐ARTEX_10:0_, Tri‐ARTEX_8:2_, and Tri‐ARTEX_6:4_. F) Hematoxylin and eosin (H&E)–stained images of the inflamed joints in each treatment group. G) Histological scores of synovial inflammation, hyperplasia, pannus formation, and bone/cartilage erosion. The histological scores were obtained from the H&E‐staining images in panel (F). Data are represented in mean ± SEM (n = 7). ns: *p* > 0.05, ^*^: *p* ≤ 0.05, ^**^: *p* ≤ 0.01, ^***^: *p* ≤ 0.001, ^****^: *p* ≤ 0.0001 versus CE+AntagomiR155; NS: *p* > 0.05, #: *p* ≤ 0.05 versus Tri‐ARTEX_8:2_. H) Quantification of pro‐ and anti‐inflammatory cytokines in the blood of the CIA mice. After 47 days of therapeutic monitoring, the blood samples were subjected to centrifugation in order to obtain serum. ELISA was used to analyze the cytokine levels present in the serum of each sample. I) Schematic representation of M1‐to‐M2 repregrogramming via liposomal membrane elasticity‐mediated systemic drug delivery. Data are represented in mean ± SD (n = 5–6). ns: *p* > 0.05, ^*^: *p* ≤ 0.05, ^**^: *p* ≤ 0.01, ^***^: *p* ≤ 0.001, ^****^: *p* ≤ 0.0001 versus CIA; NS: *p* > 0.05, #: *p* ≤ 0.05, ##: *p* ≤ 0.01, ####: *p* ≤ 0.0001 versus Tri‐ARTEX_8:2_ and analyzed by one‐way ANOVA.

**Figure:**
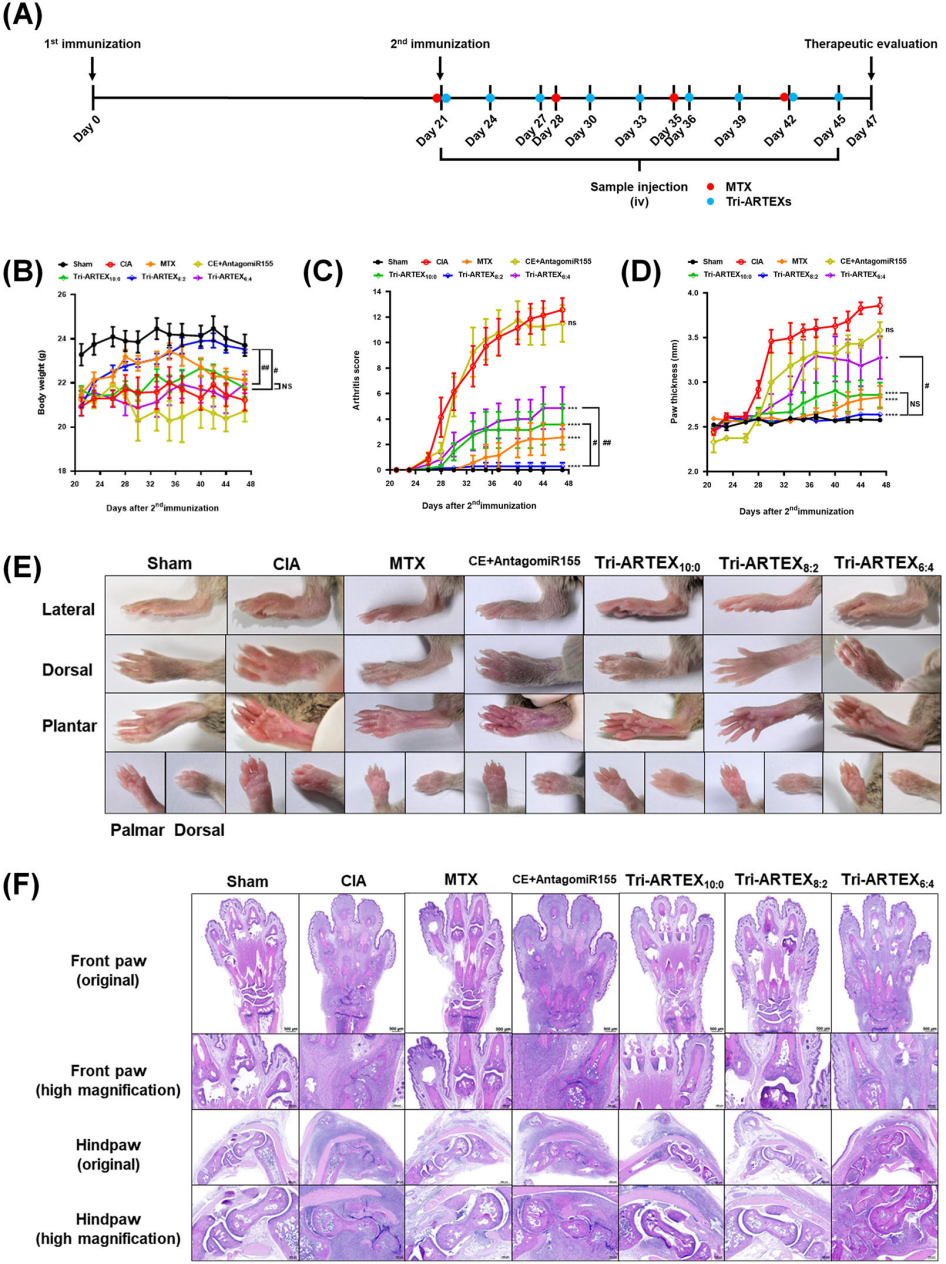

**Figure:**
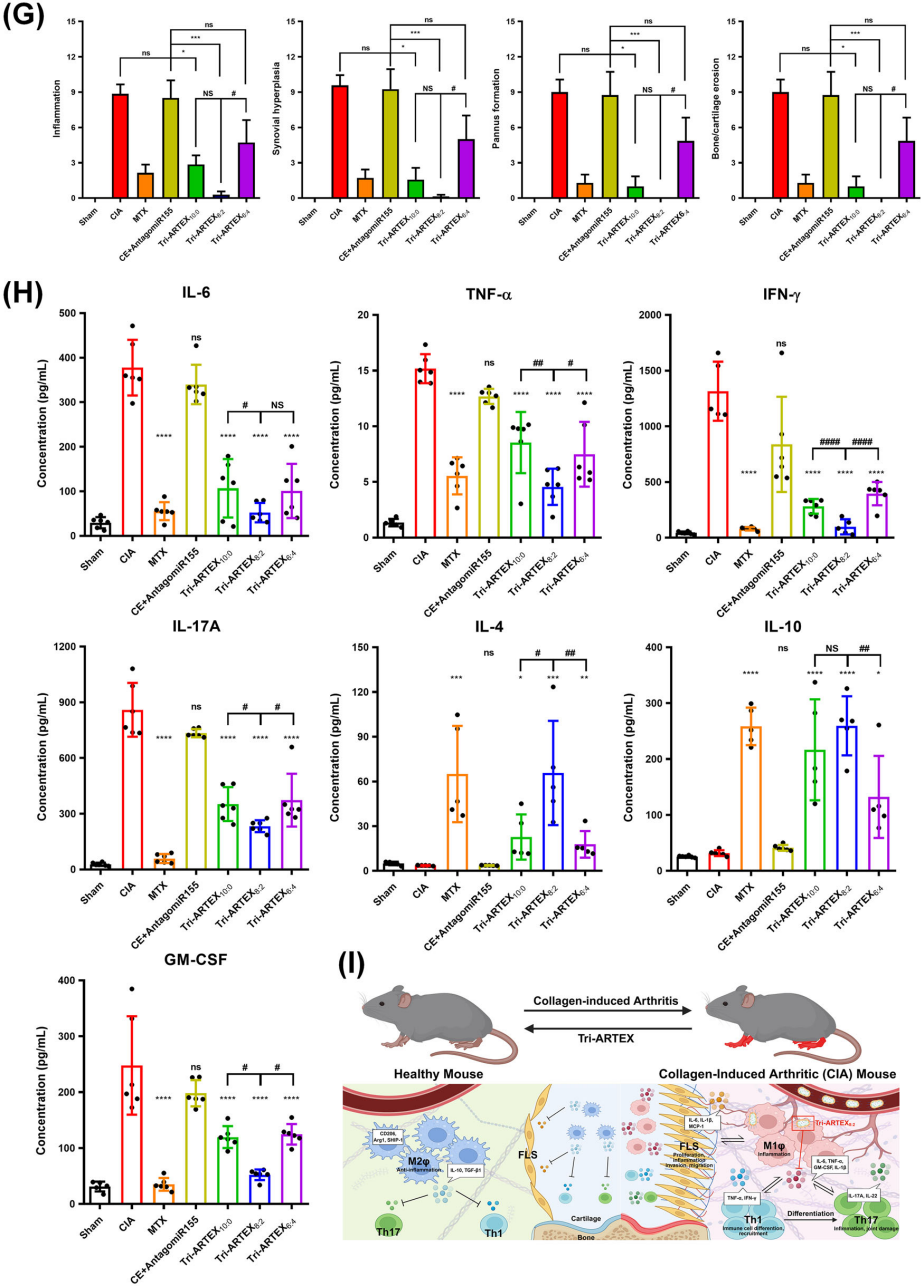

### 2.6 Synergy in Anti‐Inflammatory Effects by Tri‐ARTEX

The therapeutic efficacy of Tri‐ARTEX formulations was further assessed by analyzing pro‐ and anti‐inflammatory cytokine levels in the blood of CIA mice after 47 days of treatment. The pathological progression of RA is driven by an imbalance between pro‐inflammatory and anti‐inflammatory cytokines, with elevated levels of IL‐6, TNF‐α, IFN‐γ, GM‐CSF, and IL‐17A indicating severe inflammation. These inflammatory cytokines further stimulate macrophage type 1 (M1) polarization, fibroblast‐like synoviocyte (FLS) transformation, and T cell activation, leading to exacerbation of the disease (Figure 6I). Compared to the CIA group, all Tri‐ARTEX formulations significantly reduced the levels of pro‐inflammatory cytokines (IL‐6, TNF‐α, IFN‐γ, GM‐CSF, and IL‐17A) and enhanced the expression of anti‐inflammatory cytokines (IL‐4 and IL‐10), suggesting effective delivery of CE^[^ ^56^, ^57^ ^]^ and AntagomiR155,^[^ ^58^, ^59^ ^]^ leading to M2 macrophage polarization, inhibition of FLS proliferation, and Th17 cell inactivation (Figure 6H). Among the three formulations, Tri‐ARTEX_8:2_ exhibited the most significant anti‐inflammatory effects, which were comparable to the conventional RA treatment with MTX. The Tri‐ARTEX_8:2_ group showed the lowest production of pro‐inflammatory cytokines and the highest increase in anti‐inflammatory cytokines, demonstrating superior therapeutic efficacy. These results indicate that modulating liposomal membrane elasticity is crucial for optimizing the anti‐inflammatory effects of Tri‐ARTEX in RA treatment. Tri‐ARTEX_8:2_ with intermediate membrane elasticity displayed the most effective modulation of cytokine levels, suggesting its potential as a promising therapeutic platform for RA. To evaluate the contribution of AntagomiR155 to the therapeutic effects, we further observed in vitro reprogramming efficacy in M1‐polarized RAW264.7 macrophages. Treatment with either AntagomiR155‐loaded Tri‐LIP or CE‐loaded Tri‐LIP led to the suppression of M1 cytokine expression and promoted M1‐to‐M2 reprogramming as evidenced by increased mRNA expressions of SHIP‐1, CD206, and Arg1, comparable to levels in M2 control (Figure S9, Supporting Information). It should be noted that the simultaneous delivery of AntagomiR155 and CE by Tri‐ARTEX resulted in synergistic effects with a significantly higher level of reduction in pro‐inflammatory cytokine expression and an increase in anti‐inflammatory cytokine expression. These findings support the mechanistic role of miR‐155 inhibition in M1‐to‐M2 transition and provide a cellular basis for the cytokine modulation observed in CIA mice treated with Tri‐ARTEX formulations.

## 3 Discussion

The Tri‐ARTEX system is inspired by EVs, which have been considered as potential cell‐free therapeutics for various diseases, including RA.^[^ ^15^, ^16^, ^60^ ^]^ EVs are cell‐derived, lipid‐based nanoparticles that are secreted by most cells and reflect the biological characteristics of their donor cells. EVs control the biological responses of recipient cells by delivering bioactive molecules such as lipids, cytosolic proteins, and nucleic acids from donor cells. They can encapsulate small drugs and biological macromolecules, and their surface modification can improve systemic circulation and cellular uptake at target tissues.^[^ ^17^, ^18^, ^61^, ^62^ ^]^ However, the additional encapsulation of active ingredients and membrane surface engineering may compromise the structural integrity of natural EVs, leading to low yield, reduced stability, and limited in vivo performance.^[^ ^21^, ^22^ ^]^ To address these limitations, we employed a lipid/polymer hybrid vehicle incorporating an amphiphilic triblock copolymer, PEO‐*b*‐PCL‐*b*‐PEO, to modulate membrane elasticity and improve in vivo stability. The development of Tri‐ARTEX, synthesized using low‐cost synthetic lipids and polymers, represents a significant advancement in cost‐effective and controlled production processes. This study introduces simplified methods to produce Tri‐ARTEX through simple thin‐film hydration. The technique ensures consistent reproducibility across different batches, enhancing the reliability and uniformity of Tri‐ARTEX production. Traditional preparation of EVs often encounters challenges such as contamination with genetic material, requiring rigorous purification processes including serial extrusion, density‐gradient ultracentrifugation, and nuclease treatment.^[^ ^63^, ^64^, ^65^ ^]^ These extensive processes are labor‐intensive, time‐consuming, and costly. By contrast, Tri‐ARTEX preparation utilizes an innovative method of osmotic gradient isolation of cytosolic extracts from stem cells, effectively minimizing the risk of genetic material contamination and simplifying the purification process. By reducing the need for extensive purification steps, this approach enhances the scalability and cost‐effectiveness of Tri‐ARTEX production. Cytosolic extracts of stem cells (CE)^[^ ^56^, ^57^ ^]^ and AntagomiR155^[^ ^58^, ^59^ ^]^ were co‐encapsulated in Tri‐ARTEX for their immunosuppressive and anti‐inflammatory effects, making them ideal candidates as active ingredients for RA therapy.

The concept of mechanobiological engineering, which involves modifying the mechanical properties to achieve different or improved biological consequences, underpins our approach. Subtle changes in membrane mechanical properties can profoundly affect the therapeutic efficacy of Tri‐ARTEXs for RA treatment. We hypothesized that modulation of systemic biodistribution and anti‐inflammatory effects of the liposomes could be attributed to the fine‐tuning of liposomal membrane elasticity, as controlled by polymer fraction. The lipid/polymer hybrid system showed increased liposomal membrane elasticity with a greater PEO‐*b*‐PCL‐*b*‐PEO fraction. The PEO‐*b*‐PCL‐*b*‐PEO copolymer exerts a bilayer‐tightening effect^[^ ^33^ ^]^ because the single hydrophobic chain of the PCL block organizes the disordered packing arrangement between unsaturated chains of DOTAP and DOPE^[^ ^66^, ^67^ ^]^, thus increasing membrane elasticity.

Besides improved mechanical properties and therapeutic performance, this polymer modification also plays a critical role in enhancing the biocompatibility of the Tri‐ARTEX system. DOTAP liposomes, while extensively investigated cationic lipids for gene and drug delivery, are known to cause rapid systemic clearance at high doses due to their strong positive surface charge.^[^ ^68^, ^69^, ^70^ ^]^ However, DOTAP formulations have shown their tolerability and therapeutic efficacy in clinical trials, including intranasal administration in cystic fibrosis patients^[^ ^71^ ^]^ and intravenous administration in EndoTAG‐1 for cancer therapy.^[^ ^72^, ^73^, ^74^, ^75^, ^76^ ^]^ To further improve biocompatibility, we incorporated the amphiphilic triblock copolymer PEO‐*b*‐PCL‐*b*‐PEO, a well‐established biocompatible polymer used in various drug formulations due to its amphiphilic nature^[^ ^77^, ^78^, ^79^ ^]^ and biodegradability.^[^ ^80^ ^]^ Therefore, the combination of DOTAP liposomes with PEO‐b‐PCL‐b‐PEO not only enhances formulation stability and biocompatibility but also reinforces the therapeutic potential of the Tri‐ARTEX platform as a targeted and safe delivery system for RA therapy.

Tuning liposomal membrane elasticity with PEO‐*b*‐PCL‐*b*‐PEO also regulated systemic biodistribution, retention in inflamed joints, and anti‐inflammatory effects in the CIA model. The Tri‐ARTEX_8:2_ with intermediate elasticity demonstrated prolonged systemic circulation time, enhanced joint accumulation in CIA mice, and down‐regulated RA‐related cytokines. In contrast, softer Tri‐ARTEX_10:0_ and more rigid Tri‐ARTEX_6:4_ showed shorter joint retention times and comparatively moderate anti‐inflammatory effects. Recent reports indicate that controlling liposomal membrane elasticity can achieve selective targeting to different disease sites, prolong retention time in blood circulation, and increase drug accumulation in target cells, thereby improving therapeutic efficacy^[^ ^66^, ^81^, ^82^, ^83^, ^84^ ^]^. By varying the acyl chain type within the phospholipid composition, liposomes with low elastic moduli can selectively target healthy cells, while those with higher elastic moduli are selectively accumulated in cancer cells with higher metastatic potential.^[^ ^85^ ^]^ Other studies have modulated mechanical properties using cholesterol content^[^ ^81^, ^86^, ^87^ ^]^ and varying the length and degree of saturation of phospholipid acyl chains,^[^ ^66^ ^]^ demonstrating that elasticity regulation influences the pharmacokinetics (PK) and pharmacodynamics (PD) of tumor‐targeted drug delivery. These findings underscore the profound influence of liposomal membrane elasticity on modulating PK/PD profiles. Mechanobiological engineering provides a framework for understanding how mechanical modifications in liposomal drug delivery systems can dictate the biological fate of nanoparticles, leading to enhanced therapeutic outcomes.

Despite extensive studies on liposomal membrane elasticity in cancer drug delivery,^[^ ^66^, ^81^, ^82^ ^]^ similar investigations in the exact mechanism by which the property affects the liposome's half‐life in systemic circulation and anti‐inflammatory effects in autoimmune diseases such as RA remain scarce. Both RA and solid tumors share characteristics of abnormal extracellular matrix (ECM) with leaky vasculature, lack of functional lymphatic drainage, and a fibrotic network, manifested through ELIVIS and EPR effects, respectively. Inspired by tumor targeting strategies, we propose that modulating liposomal membrane elasticity offers a promising strategy to overcome analogous biological barriers in RA. The mechanical properties of liposomes, particularly liposomal membrane elasticity, are emerging as critical parameters influencing their interaction with pathological ECM. As previously described by Dai et al.,^[^ ^66^ ^]^ soft liposomes would tend to be trapped in the fibrotic mesh network due to excessive deformation, increasing the contact area between the liposomes and the ECM, which leads to physical entanglement or increased adhesion. Conversely, rigid liposomes could retain their spherical structure during ECM diffusion, showing lower diffusivity compared to semi‐elastic liposomes. Semi‐elastic liposomes with intermediate elasticity would adapt their shape during diffusion, enabling rotational movement around the fibrotic network and facilitating translational transport to avoid entrapment in the ECM. Therefore, controlling liposomal membrane elasticity may play a crucial role in ECM diffusion, leading to deeper ECM penetration and enhanced cellular uptake. These results suggest that fine‐tuning liposomal membrane elasticity is a determining factor in designing effective systemic drug delivery carriers for RA treatment.

## 4 Conclusion

In this study, we developed a novel lipid/polymer hybrid vehicle, Tri‐ARTEX, that incorporates the triblock copolymer PEO‐*b*‐PCL‐*b*‐PEO to modulate liposomal membrane elasticity for improved therapeutic efficacy in rheumatoid arthritis (RA). Inspired by EVs, Tri‐ARTEX mimics the cell‐modulating functions of EVs by incorporating CE and AntagomiR155 for their immunosuppressive and anti‐inflammatory effects. Our findings demonstrate that fine‐tuning liposomal membrane elasticity is crucial for optimizing systemic biodistribution, joint accumulation, and anti‐inflammatory effects. The Tri‐ARTEX_8:2_ formulation with intermediate elasticity showed prolonged systemic circulation, improved joint targeting, and significant down‐regulation of RA‐related cytokines, outperforming both softer and more rigid counterparts. This study highlights the potential of mechanobiologically engineered EV mimics as effective systemic drug delivery carriers for RA treatment, offering new insights into the design of advanced therapeutic platforms that address the unique pathophysiological challenges of RA.

## 5 Experimental Section

### Materials

1,2‐dioleoyl‐3‐trimethylammonium‐propane (DOTAP) chloride salt, 1,2‐dioleoyl‐sn‐glycero‐3‐phosphoethanolamine (DOPE), and 1,2‐dioleoyl‐sn‐glycero‐3‐phosphoethanolamine‐N‐[methoxy(polyethylene glycol)‐5000] (PEG5k‐DOPE, 18:1 PEG5000 PE) ammonium salt were purchased from Avanti Polar Lipids Inc. (Alabaster, AL, USA). The amphiphilic triblock copolymer, PEO‐*b*‐PCL‐*b*‐PEO (Mw ∼ 20,000 g mol^−1^, Mw of blocks 5000‐10000‐5000 g mol^−1^), was kindly supplied by SK Bioland (Korea). Cyanine5.5 (Cy5.5) NHS ester was purchased from Lumiprobe (Cockeysville, MD, USA). Texas red‐1,2‐dihexadecanoyl‐sn‐glycero‐3phosphoethanolamine (Texas Red‐DHPE) and 3‐(4,5‐dimethylthiazol‐2‐yl)‐2,5‐diphenyl tetrazolium bromide for (MTT) assay were purchased from Sigma‐Aldrich (St. Louis, MO, USA). Raw 264.7 cells were purchased from the Korean Cell Line Bank (Seoul, Korea). StemPro^TM^ human adipose‐derived stem cells (ADSC), MesenPRO RS^TM^ basal medium, and MesenPRO RS^TM^ growth supplement were purchased from Invitrogen (Waltham, MA, USA). Animal‐free recombinant human epidermal growth factor (hEGF) and human fibroblast growth factor‐basic (hFGF‐b) were purchased from PeporoTech (Waltham, MA, USA). Lipopolysaccharides (LPS) from Escherichiacoli were purchased from Sigma–Aldrich (St. Louis, MO, USA), and recombinant murine interferon‐gamma (IFN‐γ) was obtained from Sino Biological (Beijing, China). Fetal bovine serum (FBS), 200 mm L‐glutamine, 10,000 U mL^−1^ penicillin‐streptomycin (Pen‐Strep), and TrypLE^TM^ Express were purchased from Gibco (Waltham, MA, USA). Dulbecco's modified Eagle's medium (DMEM) and Dulbecco's PBS (DPBS) were purchased from WelGENE (Gyeongsan, Korea). cOmplete^TM^, EDTA‐free protease inhibitor cocktail was purchased from Roche (Basel, Swiss). Water was purified using an Alto™ I Type 1 Ultrapure Water System from Avidity Science (Waterford, WI, USA). Formic acid, lithium carbonate, ammonium hydroxide, and ammonium oxalate were purchased from Sigma‐Aldrich (St. Louis, MO, USA). All other chemicals and solvents were of ACS reagent grade and used without further purification. miRNA‐155 inhibitor (AntagomiR155) was customized from Bioneer (Daejeon, Korea). The sequence of AntagomiR155 is ACCCCUAUCACAAUUAGCAUUAA.

### Synthesis of Cy5.5‐Conjugated DOPE

Cy5.5‐conjugated DOPE was synthesized by using the ester‐amine reaction according to standard protocols.^[^ ^88^ ^]^ Briefly, Cy5.5‐NHS‐ester was dissolved in DMF (1 mg mL^−1^), and DOPE was dissolved in chloroform (1 mg mL^−1^). Then, the two molecules were mixed in a ratio of Cy5.5‐NHS‐ester/DOPE = 3/1 (v/v) and stirred for 4 h at room temperature. Next, chloroform was evaporated with a rotary evaporator for 1 h, and the remaining DMF and unconjugated DOPE lipids were eliminated by dialysis using a 1000 MWCO membrane in deionized water (DW) for 24 h. Finally, Cy5.5‐conjugated DOPE in the dialysis bag was transferred to a vial and freeze‐dried overnight. The final product was stored at ‐20 °C and used by first dissolving the lyophilized material in chloroform for the preparation of Cy5.5‐labeled Tri‐LIPs.

### Cell Culture

ADSCs were thawed and expanded in Complete MesenPRO RS^TM^ medium containing MesenPRO RS^TM^ growth supplement and 2 mm L‐glutamine. Passage #3 and #4 ADSCs were cultured in the complete DMEM supplemented with 10% FBS, 1% Pen‐Strep, 20 ng m^−1^L hEGF, and 20 ng mL^−1^ hFGF‐b at 37 °C in a humidified 5% CO_2_ atmosphere. A murine macrophage cell line, Raw 264.7 (Korea Cell Line Bank, Seoul, Korea), was cultured in DMEM supplemented with 10% FBS and 1% Pen‐Strep at 37 °C in a humidified 5% CO_2_ atmosphere.

### Preparation of Adipose‐Derived Stem Cell Extract (CE)

Passage #5 or #6 ADSCs were used for CE preparation. ADSCs were detached by TrypLE^TM^ Express and centrifuged. The cell pellet was washed three times with DPBS to eliminate any remaining FBS, and 2 × 10^6^ cells were gently resuspended in 437.5 µL of 0.2 µm‐filtered DW to induce osmotic lysis. The cell suspension was then lysed on ice for 30 min to ensure complete cell lysis by osmotic shock without additional mechanical or chemical methods. Afterward, 62.5 µL of 8x PBS was added to restore isotonicity of the cell lysate. The cell lysate was centrifuged at 1,100 × *g* for 10 min at 4 °C to remove cell debris and genetic contaminants, followed by a second round of centrifugation at ≥ 14,000 × *g* for 10 min at 4 °C to further purify the CE. The resulting supernatant was collected as the CE and stored at ‐80°C until use. The amount of the prepared CE was quantified by using the Pierce™ BCA Protein Assay Kit from Thermo Fisher Scientific (Waltham, MA, USA).

### Preparation of ADSC‐Derived EVs

Passage #5 or #6 ADSCs were used for EV preparation. ADSCs were cultured in complete DMEM at 80% confluency. The cells were washed twice with DPBS, and then the culture medium was changed to FBS‐free DMEM. After 24‐h starvation, the supernatant media were filtered through 0.22‐µm filters to remove cell debris from the medium. The filtered sample was then purified by centrifugation using an Amicon® Ultra (AU) filter (100k MWCO) to remove other large‐molecule proteins and to concentrate the EV solution. Each supernatant was collected and serially centrifuged at 300 × g at 4 °C for 10 min, 2000 × g at 4 °C for 10 min, and 10 000 × g at 4 °C for 30 min. The final supernatant was centrifuged by the ultra‐centrifugation (Optima XE‐90, Beckman Coulter, Brea, CA, USA) at 120,000 × *g* for 16 h at 4 °C. The supernatant was discarded, and EVs in the pellet were used for further experiments.

### Preparation and Characterizations of Tri‐LIPs and Tri‐ARTEXs

To prepare Tri‐LIPs, lipids (DOPE/DOTAP = 4/5, w/w) and PEO‐*b*‐PCL‐*b*‐PEO were fully dissolved in chloroform in different ratios (10:0, 8:2, 6:4) and then collected in a 50 mL round‐bottom flask. Chloroform solvent was evaporated using a rotary evaporator at room temperature for 1 h to obtain a thin lipid/polymer hybrid film. The film was hydrated with PBS in a bath sonicator for 1 h, and then the hydrated lipid/polymer suspension was subjected to ultrasonication set at 30% of maximum amplitude and underwent 20 s/10 s on/off intervals for 3 min. For Cy5.5‐labeled and Texas Red‐labeled Tri‐LIPs, Cy5.5‐DOPE and Texas Red‐DHPE were added in chloroform, respectively, before the evaporation step. Thermal properties of Tri‐LIPs were analyzed by using differential scanning calorimetry (Seiko DSC7020, SEIKO Instruments, Tokyo, Japan). For DSC analysis, sample films (8 mg) made with DOPE/DOTAP, PEO‐*b*‐PCL‐*b*‐PEO, and the appropriate mixture of DOPE/DOTAP and PEO‐*b*‐PCL‐*b*‐PEO were loaded into a high‐volume pan. The heating rate was adjusted from ‐40 to 80 °C at a rate of 10 °C min^−1^.

To prepare Tri‐ARTEXs, Tri‐LIPs (1 mg mL^−1^) were used to encapsulate CE at a final concentration of 2 mg mL^−1^ and AntagomiR155 at 21 µm and sonicated in an ice bath for 15 min using 3‐second on/3‐second off cycles. After the loading step, Tri‐ARTEXs were centrifuged at 22,000 x g at 4 °C for 6 min to remove unloaded CE and AntagomiR155 in the supernatant. The collected supernatant was used to determine the drug loading content (LC) and encapsulation efficiency (EE) of CE and AntagomiR155. The resulting Tri‐ARTEXs in the pellet were resuspended in 1 mL of PBS. LC and EE were measured using the Pierce™ BCA Protein Assay Kit and Quanti‐iT^TM^ microRNA Assay Kit from Thermo Fisher Scientific (Waltham, MA, USA). LC and EE were calculated according to the following equations (1) and (2), respectively:

(1) $$\mathrm{LC}\;\left(\%\right)=\frac{A_{\mathrm{loaded}}}{A_{\mathrm{loaded}\;}+A_{\mathrm{Tri}-\mathrm{LIP}}}\times 100$$

(2) $$\mathrm{EE}\;\left(\%\right)=\frac{A_{\mathrm{total}}-A_{\mathrm{supernatant}}}{A_{\mathrm{total}}}\times 100$$

where A_total_ is the total amount of CE or AntagomiR155 added to the Tri‐LIP formulation during sample preparation, A_supernatant_ is the amount of unloaded CE or AntagomiR155 in the supernatant, and A_loaded_ is the amount of loaded CE or AntagomiR155 that is determined by subtracting A_supernatant_ from A_total_.

The particle size, polydispersity index (PDI), and zeta potential of Tri‐LIPs and Tri‐ARTEXs were measured by dynamic light scattering (DLS) and electrophoretic mobility measurements by using a Zetasizer Nano ZS90 system (Malvern Instruments, Worcestershire, UK) at 25 °C. For DLS measurements, samples were exposed to a He‐Ne red laser with a 633‐nm wavelength that was set at a scattering angle of 90°. Three measurement replicates were recorded per sample.

The morphologies of both Tri‐LIPs and Tri‐ARTEXs were imaged using transmission electron microscopy (TEM; JEOL‐2100F instrument, JEOL Ltd., Tokyo, Japan) at an accelerating voltage of 200 kV. For sample preparation, the Tri‐LIPs and Tri‐ARTEXs were placed onto a 200‐mesh carbon‐film‐coated grid without drying, negatively stained with 2% uranyl acetate, and rinsed with deionized water.

### QCM‐D Measurements

For QCM‐D analysis, Tri‐LIPs were prepared with 10 mm Tris [pH 7.5] with 150 mm NaCl according to the same protocol described above. The QCM‐D experiments were performed using a Q‐Sense E4 instrument (Biolin Scientific AB, Stockholm, Sweden), as previously described.^[^ ^50^ ^]^ The QCM‐D sensor chips had a fundamental frequency of 5 MHz and a sputter‐coated, 50 nm thick titania layer (model no. QSX 310, Biolin Scientific AB). Prior to the experiment, the sensor chip surfaces were extensively rinsed with water and ethanol, followed by drying and then oxygen plasma treatment for 1 min in a CUTE‐1MPR machine (Femto Science Inc., Hwaseong, Republic of Korea). All liquid samples were injected into the QCM‐D measurement chambers by a peristaltic pump (Reglo Digital, Ismatec, Glattbrugg, Switzerland) and the volumetric flow rate was fixed at 100 µL min^−1^. The QSoft software program (Biolin Scientific AB) was used for data collection at multiple odd overtones, and all data are presented in normalized form after dividing by the overtone number accordingly. All presented QCM‐D data were collected at the 5th overtone, while the extended Voigt viscoelastic model was applied to analyze adlayer thickness and viscosity from data obtained at the 3rd, 5th, and 7th overtones within the QTools software package (Biolin Scientific AB). The effective adlayer thickness and viscosity were obtained by model fitting based on constraining the bulk aqueous solution to have a uniform density and viscosity of 1000 kg m^−3^ and 0.001 Pa·s, respectively. All reported QCM‐D data and modeling results are from three independent replicates.

### Western Blotting

EVs and ARTEX were lysed by 1x RIPA buffer containing 1x cOmplete EDTA‐free protease inhibitor cocktails, followed by centrifugation to remove the lysate. Total protein concentration was determined by BCA protein assay. Protein samples were separated by SDS‐polyacrylamide gel electrophoresis, transferred to polyvinylidene difluoride membrane, and blocked with 5% skim milk buffer. The membranes were incubated overnight with primary antibodies, followed by incubation with the corresponding secondary antibodies at room temperature. The ALIX primary antibody was obtained from Abcam (Camb, UK), while CD63 and Hsp70 antibodies were included in the ExoAb antibody kit (System Biosciences, CA, USA). An electrochemiluminescence chromogenic substrate was applied to visualize the target bands, and imaging was performed using a ChemiDoc system (Bio‐Rad, USA).

### Evaluation of Cytotoxicity and Cellular Uptake of Tri‐LIPs

The cell viability of Tri‐LIPs was determined by using the MTT assay. Raw 264.7 cells were seeded in a 96‐well plate at a density of 1 × 10^4^ cells per well and incubated for 24 h. Next, the cells were treated with different concentrations of Tri‐LIPs for another 24 h. Then, the cells were washed with PBS, and MTT solution diluted in DMEM was added to each well and incubated for 2 h at 37 °C. Finally, sample absorbance was measured at 450 nm using a microplate reader (SPARK, Tecan, Switzerland), and cell viability was calculated accordingly. To measure the cellular uptake of Tri‐LIPs, 1 × 10^4^ Raw 264.7 cells were seeded in a 96‐well plate and incubated for 24 h. Texas Red‐incorporated Tri‐LIPs were diluted using cell culture medium and treated to the cells for different time periods (0, 1, 2, 3, 4, 5, and 6 h) After washing the dispersed sample twice with PBS, fluorescence was measured at 535/595 nm (excitation/emission) using a microplate reader. To visualize the internalized Tri‐LIPs in the Raw 264.7 cells, 1 × 10^4^ cells were seeded in confocal dishes and incubated for 24 h. Next, the cells were treated with Texas Red‐DHPE incorporated‐Tri‐LIPs and incubated for 4 h at 37 °C. The cells were then fixed with 4% paraformaldehyde for 10 min and stained with 1 µg mL^−1^ DAPI for 5 min at room temperature. Fluorescence images were obtained by using a confocal laser scanning microscope (CLSM, TCS SP8 HyVolution, Leica).

### Tri‐ARTEX‐Guided Reprogramming of M1 Macrophages

Raw264.7 cells were seeded in 6‐well plates at a density of 3 x 10^5^ cells per well in 2 mL of complete DMEM supplemented with 500 ng mL^−1^ LPS and 20 ng mL^−1^ IFN‐γ at 37 °C in a humidified 5% CO_2_ atmosphere for 24 h to induce M1 polarization. After polarization, the cells were transfected in serum‐free DMEM with one of the following formulations: Tri‐LIP_8:2_, AntagomiRcontrol@Tri‐LIP_8:2_, AntagomiR155@Tri‐LIP_8:2_, Tri‐ARTEX_8:2_, AntagomiR155@Tri‐ARTEX_8:_2. Final concentrations were 20 µg mL^−1^ CE, 0.21 µm AntagomiR155 or control, and 10 µg mL^−1^ Tri‐LIP_8:2_. Transfection was conducted at 37 °C for 6 h. Subsequently, the cells were washed with PBS and post‐incubated in complete DMEM for 36 h. Reprogramming efficacy was assessed by measuring mRNA levels of M1 pro‐inflammatory markers (TNF‐α, IL‐6, GM‐CSF, and IL‐1β) and M2 anti‐inflammatory markers (CD206, Arg1, and SHIP‐1), using quantitative Reverse Transcription PCR (qRT‐PCR). Total RNA was extracted using QIAshedder and RNeasy kits (Qiagen Inc., Hilden, Germany) according to the manufacturer's instructions. RNA concentration was determined using Nanodrop 2000 (Thermo Scientific, Waltham, MA, USA). cDNA synthesis and real‐time PCR were performed using the TOPreal™ SYBR Green RT‐qPCR Kit (Enzynomics, Daejeon, Korea) according to the manufacturer's instructions. All reactions were performed in triplicate in a 20 µL volume. qRT‐PCR data were analyzed using CFX Manager^TM^ software (Bio‐Rad Laboratories, Hercules, CA, USA). The primer sequences are listed in Table S3 (Supporting Information).

### Induction of Collagen‐Induced Arthritis (CIA) Model

All animal experiments were conducted in compliance with relevant laws and institutional guidelines of Sungkyunkwan University and were approved by the Institutional Committee (SKKUIACUC2022‐07‐35‐1). Six‐week‐old male DBA1/J mice (20 ± 2 g) were purchased from Central Lab Animal Inc. (Seoul, Korea) and acclimatized under natural 12 h light/dark cycles at 25 °C and 55% humidity. To induce the murine CIA model, the following steps were taken according to the CIA modeling protocol from Brand et al.:^[^ ^89^ ^]^ on Day 0, seven‐week‐old DBA1/J mice were intradermally injected in the tail with 200 µg bovine type II collagen (2 mg mL^−1^, Chondrex, Redmond, WA, USA) that was mixed with 100 µL of 4 mg mL^−1^ Complete Freund's Adjuvant. On day 21, the mice received a booster immunization of type II collagen mixed with Incomplete Freund's Adjuvant and were evaluated every second or third day for arthritis incidence. Each paw was evaluated on a scale of 0‐4, which was scored as follows: 0 indicating normal, no inflammation or redness; 1 indicating mild redness and swelling in one or two digits with no apparent swelling of paw or ankle; 2 indicating moderate redness and swelling in more than three digits without paw swelling or mild swelling of entire paw without swelling in all digits; 3 indicating severe swelling of entire paw; 4 indicating the most severe swelling and inflammation of the entire paw and all digits or ankylosed paw and toes, and the mouse could not grip the wire top of the cage. The scores for all paws were added up for each mouse, with a maximum score of 16.

### in vivo Biodistribution of Cy5.5‐Labeled Tri‐LIPs

On day 42 after CIA induction, the sham treatment (PBS) or one of three different cy5.5‐labeled Tri‐LIP formulations (100 µg kg^−1^) was intravenously (IV) injected into the CIA mice with an arthritic score above 12. The in vivo distribution of the Tri‐LIP formulations was imaged by using an in vivo imaging system (SPECTRAL Lago X; Spectral Instruments Imaging, Inc., Tucson, AZ, USA) at different time intervals as follows: 0, 3, 9, 24, 48 h. Aura software (Spectral Instruments Imaging, Inc., Tucson, AZ, USA) was used to analyze and express all images. Mice were euthanized at 3 h after IV injection of the sample, and their major organs were excised and observed using the in vivo imaging system.

### In Vivo Therapeutic Efficacy of Tri‐ARTEXs

The mice were divided into seven groups to evaluate the therapeutic effects of Tri‐ARTEX liposomal elasticity. The sham group was IV injected with PBS. CIA mice were intravenously administered PBS (100 µL), 2 mg mL^−1^ CE and 21 µm AntagomiR155 (100 µL), 2.5 mg kg^−1^ MTX (100 µL), Tri‐ARTEX_10:0_ (180 µL), Tri‐ARTEX_8:2_ (174 µL), or Tri‐ARTEX_6:4_ (164 µL), starting on the day of the second immunization. Mice were monitored for arthritis incidence and body weight loss, and paw thickness for both right and left paws was measured using calipers every second or third day, starting on day 21. For the histological analysis, the joints were fixed with 10% buffered neutral formalin for four days and decalcified for 48 h using a 6% formic acid solution. Residual calcium was checked every 12 h during the decalcification step to avoid over‐decalcification, using equal parts of 5% ammonium hydroxide solution and 5% ammonium oxalate solution. Once the decalcification step was complete, the specimens were neutralized in 0.25% lithium carbonate solution and fixed for 48 h. The paraffin sections of each joint were then sliced (5 µm in thickness), stained with hematoxylin and eosin, and analyzed using a slide scanner (Axioscan.Z1; Carl Zeiss Microscopy GmbH, Jena, Germany). To investigate pro‐ and anti‐inflammatory cytokine levels, sham and CIA mice were sacrificed, and blood samples were collected after 47 days of therapeutic monitoring. The collected blood samples were centrifuged to obtain serum. The serum samples were then analyzed using ELISA MAX^TM^ Deluxe Set Mouse assay kits (BioLegend, San Diego, CA, USA) according to the manufacturer's instructions. Sample absorbance (450 nm) was measured using a microplate reader.

## Conflict of Interest

The authors declare no conflict of interest.

## Author Contributions

D. K. and H. B. contributed equally to this work. The manuscript was written through the contributions of D. K., H. B., J. A. J., J. W. K., and J. H. J. All authors have given approval to the final version of the manuscript. D. K. and H. B. contributed to investigation, formal analysis, data curation, visualization, and project administration. S. Y. L., M. S. L., S. L., J. L., T. N. S., and J. Y. K. all contributed to the investigation. D. K., H. B. and M. G. contributed to the revision. J. A. J., J. W. K., and J. H. J. contributed to supervision, resources, funding acquisition, and conceptualization.

## Supporting information



Supporting Information

## Data Availability

The data that support the findings of this study are available from the corresponding author upon reasonable request.
