# Supplementary material for: Mechanobiologically Engineered Mimicry of Extracellular Vesicles for Improved Systemic Biodistribution and Anti‐Inflammatory Treatment Efficacy in Rheumatoid Arthritis
Source: Adv Healthc Mater. 2025 Aug 9;14(25):2500795. doi: 10.1002/adhm.202500795 (PMC12477577; doi:10.1002/adhm.202500795)
Supplement: Supplementary file 1 — Supporting Information [file ADHM-14-0-s001.docx]

Supporting Information

Mechanobiologically Engineered Mimicry of Extracellular Vesicles for Improved Systemic Biodistribution and Anti-Inflammatory Treatment Efficacy in Rheumatoid Arthritis

Dahwun Kim, Hwira Baek, Su Yeon Lim, Min Sang Lee, Siyan Lyu, Jihyun Lee, Tun Naw Sut, Marta Gonçalves, Jeong Yi Kang, Joshua A. Jackman*, Jin Woong Kim* and Ji Hoon Jeong*

**Table S1.** Molar concentration of DOPE, DOTAP, PEG5k-DOPE, and PEO-PCL-PEO used for fabrication of Tri-LIPs.

| Sample | DOPE (μM) | DOTAP (μM) | PEO-b-PCL-b-PEO (μM) | PEG5k-DOPE (μM) | Total (μM/mg) |
| --- | --- | --- | --- | --- | --- |
| Tri-LIP_10:0_ | 435.5 | 635.6 | 0 | 40 | 1111 |
| Tri-LIP_8:2_ | 396.5 | 556.9 | 10 | 20 | 983.4 |
| Tri-LIP_6:4_ | 358.9 | 476.7 | 20 | 0 | 855.6 |


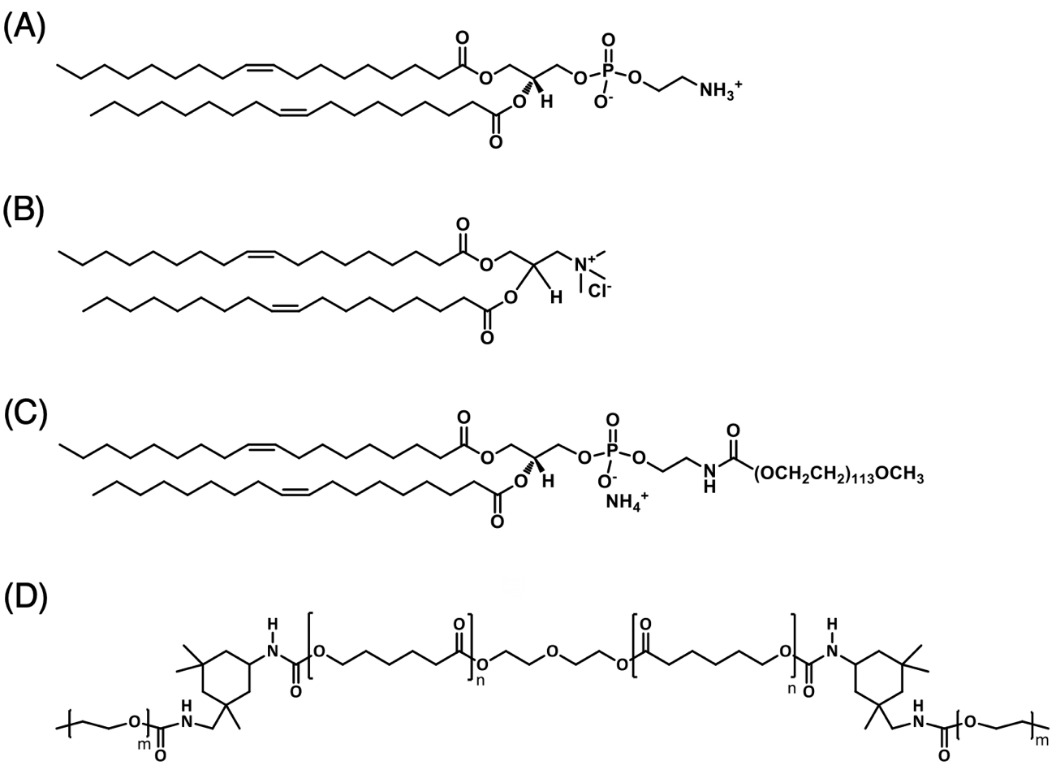


**Figure S1.** Molecular structure of (a) DOPE, (b) DOTAP, (C) PEG5k-DOPE, and (D) PEO-*b*-PCL-*b*-PEO.

**Table S2.** Mean COV for particle diameter, PDI, zeta potential, LC, and EE

|  | Particle diameter (nm)^b^ | | PDI ^b^ | | Zeta potential (mV) ^b^ | | LC (%)^b^ | | EE (%)^b^ | |
| --- | --- | --- | --- | --- | --- | --- | --- | --- | --- | --- |
|  | Tri-LIP | Tri-ARTEX | Tri-LIP | Tri-ARTEX | Tri-LIP | Tri-ARTEX | CE | AntagomiR155 | CE | AntagomiR155 |
| 10:0^a^ | 2.38 | 3.37 | 1.95 | 2.42 | 3.77 | 3.33 | 1.25 | 0.58 | 1.25 | 0.58 |
| 8:2^a^ | 2.39 | 1.56 | 3.86 | 2.42 | 3.07 | 2.17 | 1.24 | 0.86 | 1.24 | 0.86 |
| 6:4^a^ | 1.56 | 3.35 | 2.42 | 3.53 | 2.17 | 3.02 | 1.56 | 0.5 | 1.56 | 0.5 |

For three Tri-ARTEX preparations, LC, drug loading content; EE, encapsulation efficiency. ^a^Composition ratio of phospholipids (DOTAP and DOPE) vs. PEO-*b*-PCL-*b*-PEO. ^b^Data are represented as mean ± SD from six independent experiments (n=6).


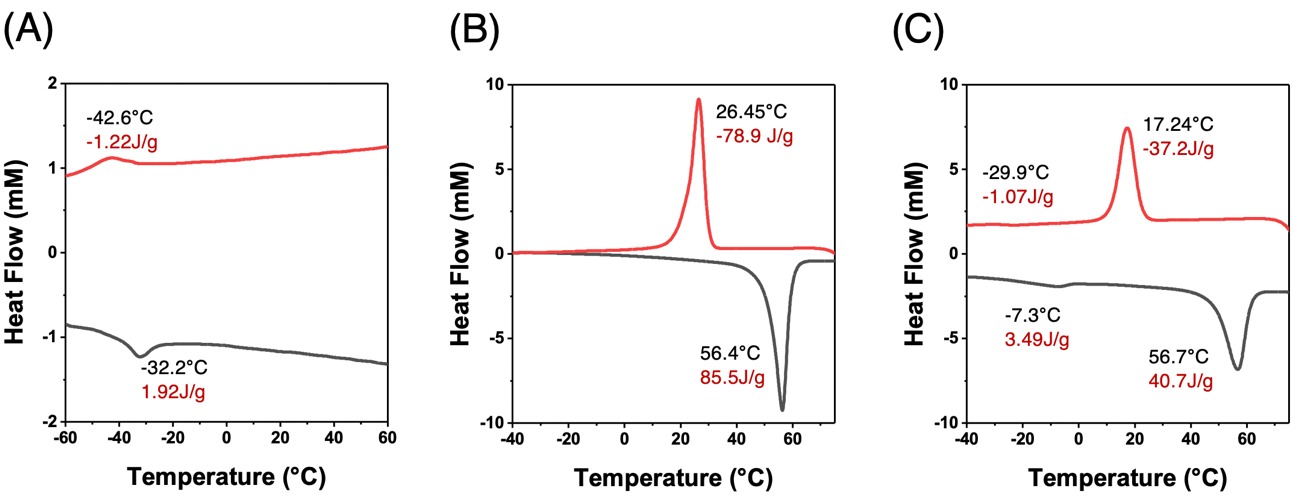


**Figure S2.** DSC thermograms of dried lipids, polymer, and lipid/polymer films: (A) DOPE/DOTAP only, (B) PEO-*b*-PCL-*b*-PEO only, (C) mixture of DOPE/DOTAP and PEO-*b*-PCL-*b*-PEO (8/2, w/w).


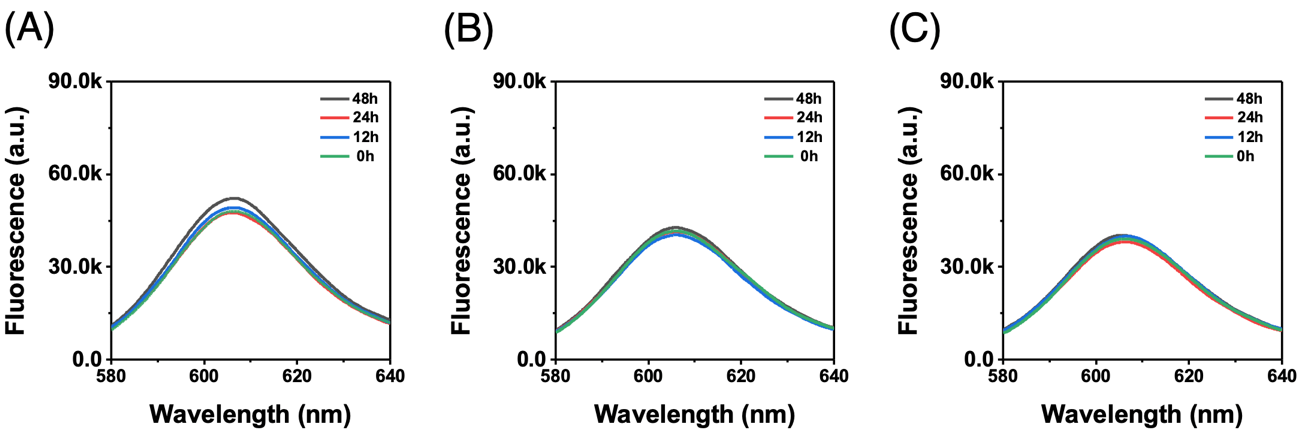


**Figure S3.** Fluorescence spectra of Texas red-loaded (A) Tri-LIP_10:0_, (B) Tri-LIP_8:2_ and (C) Tri-LIP_6:4_ after incubation with FBS/PBS (1/9, v/v) with varying incubation time at 37 °C.


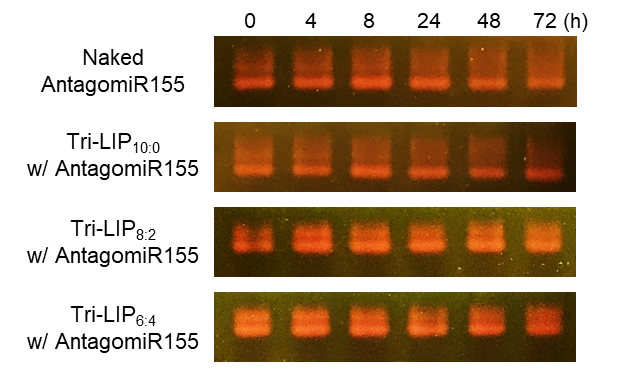


**Figure S4.** Serum stability assessment of naked AntagomiR155 and AntagomiR155-loaded Tri-LIPs via agarose gel electrophoresis. Samples were incubated in 50% serum solution at 37°C and analyzed at multiple time points (0, 4, 8, 24, 48, and 72 h).


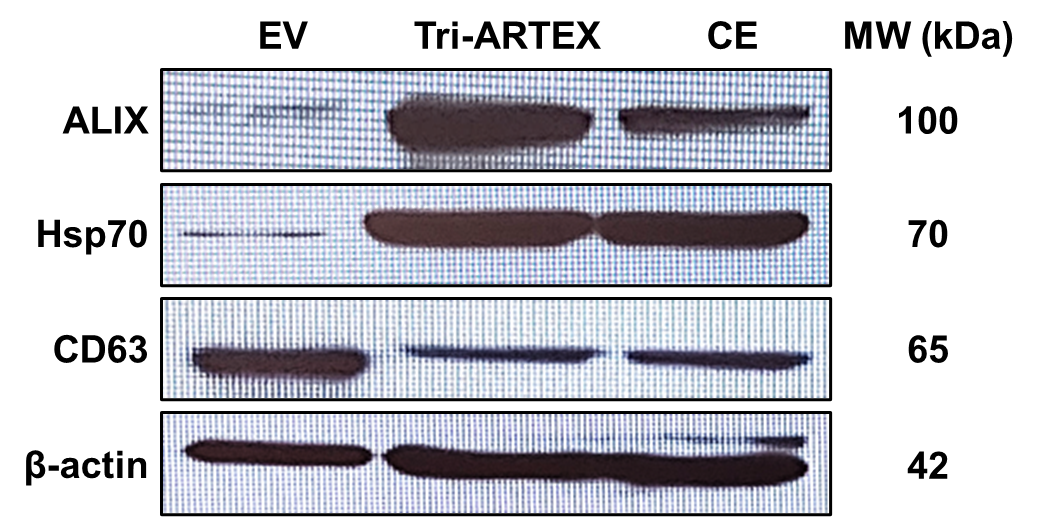


**Figure S5.** Western blotting of EV markers (ALIX, Hsp70, and CD63) in ADSC-derived EV, ARTEX, and CE. β-actin was used as a houskeeping standard. MW, molecular weight.

**
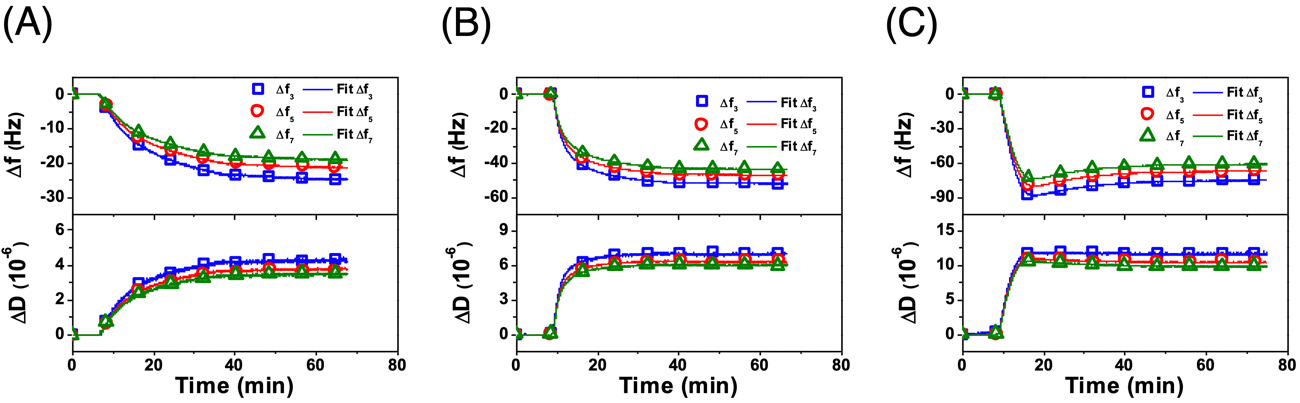
**

**Figure S6.** Viscoelastic model fitting of QCM-D measurement data. Experimental data (symbols) and model fits (lines) are presented for Tri-LIP adsorption data onto titania-coated QCM-D sensor chip surfaces. QCM-D data from the 3^rd^, 5^th^, and 7^th^ overtones were analyzed using an extended, Voigt-based viscoelastic model. The results are presented for (A) Tri-LIP_10:0_, (B) Tri-LIP_8:2_, and (C) Tri-LIP_6:4_.


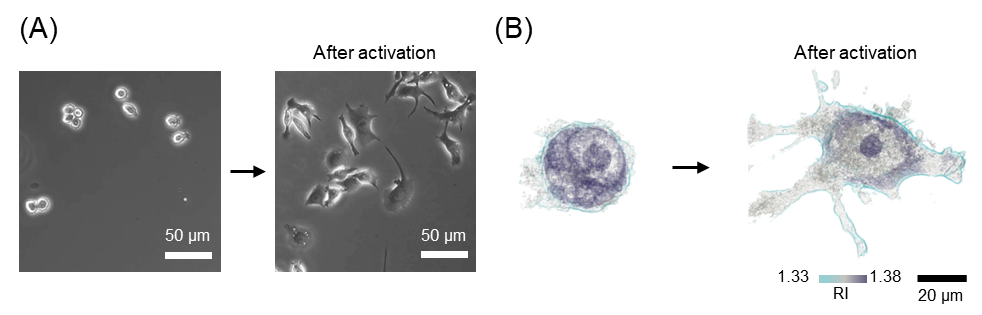


**Figure S7.** Morphological transformation of macrophages upon LPS-induced activation. (A) Brightfield microscopy images and (B) 3D holotomography of individual macrophages.


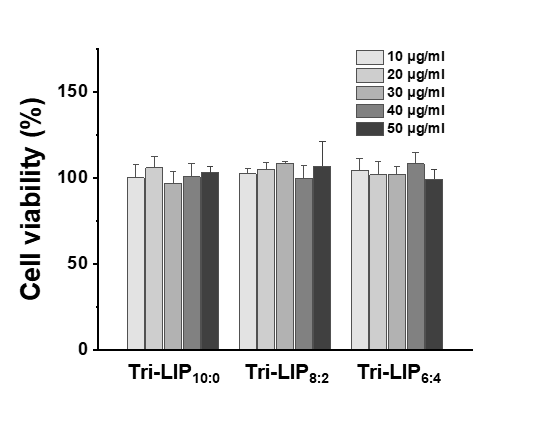


**Figure S8.** Cell viability of Raw 264.7 cells treated with different concentrations of Tri-LIPs for 24 h.


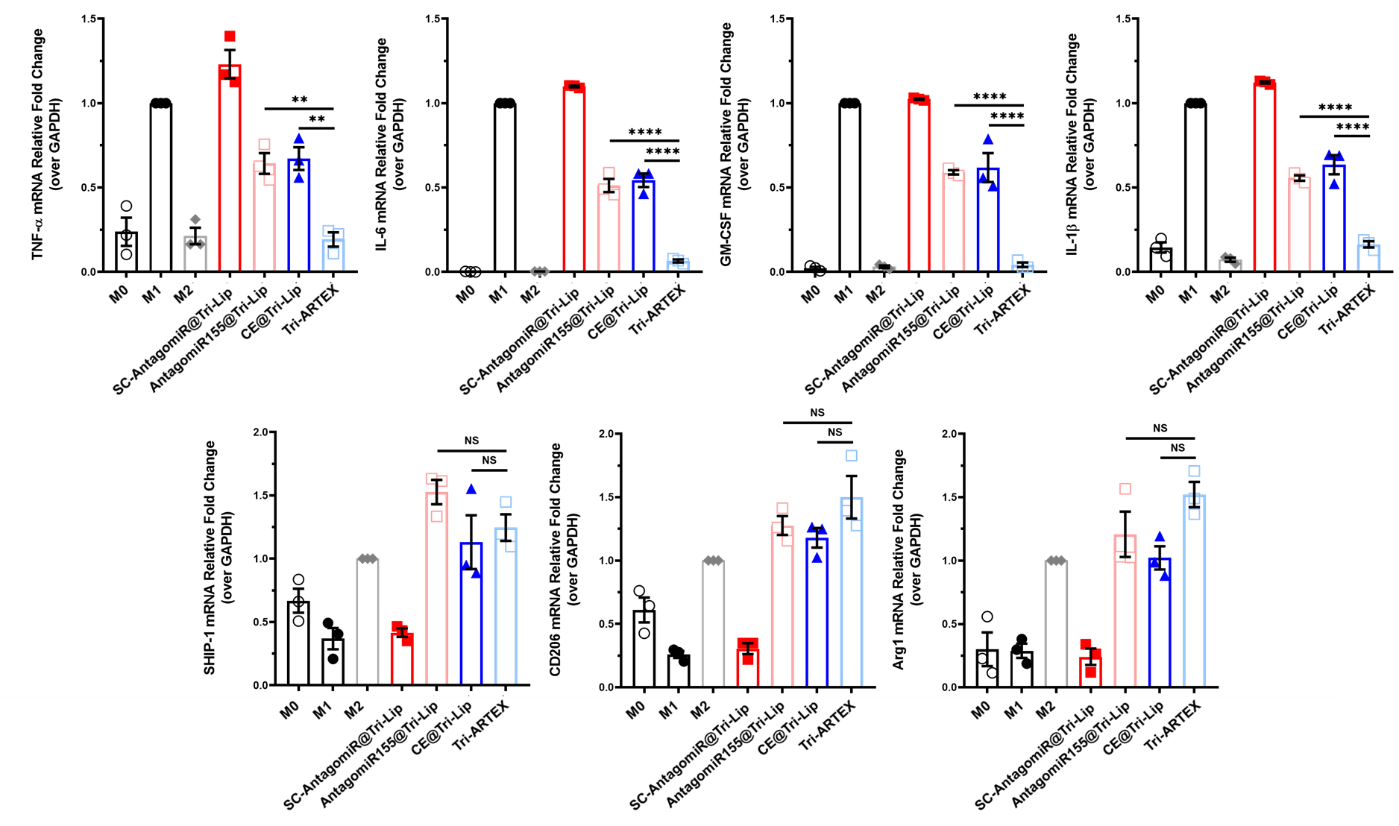


**Figure S9.** Tri-ARTEX-mediated reprogramming of M1 macrophages. Raw264.7 cells were polarized to the M1 phenotype using LPS and IFN-γ for 24 h, then the polarized cells were treated for 6 h with Tri-LIP_8:2_ containing either a scrambled control AntagomiR (SC-AntagomiR@Tri-LIP) or AntagomiR155 (AntagomiR155@Tri-LIP) or CE (CE@Tri-LIP) and Tri-ARTEX_8:2_. Reprogramming efficacy was evaluated using qRT-PCR for M1 markers (TNF-α, IL-6, GM-CSF, IL-1β) and M2 markers (CD206, Arg1, SHIP-1). GAPDH was used as a housekeeping standard. Data are represented in mean ± SEM (n = 3). NS: *p* > 0.05, **: *p* ≤ 0.01, ****: *p* ≤ 0.0001 versus Tri-ARTEX.

**Table S3.** M1 and M2 primer sequences for qRT-PCR.

| Primer Name | F/R Primer | Primer Sequence |
| --- | --- | --- |
| TNF-α | Forward | 5’-GGTGCCTATGTCTCAGCCTCTT-3’ |
|  | Reverse | 5’-GCCATAGAACTGATGAGAGGGAG-3’ |
| IL-6 | Forward | 5’-TACCACTTCACAAGTCGGAGGC-3’ |
|  | Reverse | 5’-CTGCAAGTGCATCATCGTTGTTC-3’ |
| GM-CSF | Forward | 5’-AACCTCCTGGATGACATGCCTG-3’ |
|  | Reverse | 5’-AAATTGCCCCGTAGACCCTGCT-3’ |
| IL-1β | Forward | 5’-TGGACCTTCCAGGATGAGGACA-3’ |
|  | Reverse | 5’-GTTCATCTCGGAGCCTGTAGTG-3’ |
| CD206 | Forward | 5’-GTTCACCTGGAGTGATGGTTCTC-3’ |
|  | Reverse | 5’-AGGACATGCCAG GGTCACCTTT-3’ |
| Arg1 | Forward | 5’-CATTGGCTTGCGAGACGTAGAC-3’ |
|  | Reverse | 5’-GCTGAAGGTCTCTTCCATCACC-3’ |
| SHIP-1 | Forward | 5’-CCAGGGCAAGATGAGGGAGA-3’ |
|  | Reverse | 5’-GGACCTCGGTTGGCAATGTA-3’ |
| GAPDH | Forward | 5’-CATCACTGCCACCCAGAAGACTG-3’ |
|  | Reverse | 5’-ATGCCAGTGAGCTTCCCGTTCAG-3’ |
